# Completeness of care provision and inequalities in care for people with hypertension, diabetes, and obesity: an analysis of the *Brazilian National Basic Health Units Census*

**DOI:** 10.1590/0102-311XEN239325

**Published:** 2026-11-02

**Authors:** Luiz Augusto Facchini, Elaine Tomasi, Elaine Thumé, Fúlvio Borges Nedel, Bruna Venturin, Maria del Pilar Flores-Quispe, Vitória de Oliveira Ximendes, Michele Rohde Krolow, Anaclaudia Gastal Fassa

**Affiliations:** 1 Faculdade de Medicina, Universidade Federal de Pelotas, Pelotas, Brasil.; 2 Programa de Pós-graduação em Epidemiologia, Universidade Federal de Pelotas, Pelotas, Brasil.; 3 Faculdade de Enfermagem, Universidade Federal de Pelotas, Pelotas, Brasil.; 4 Departamento de Saúde Pública, Universidade Federal de Santa Catarina, Florianópolis, Brasil.; 5 Centro de Integração de Dados e Conhecimentos para Saúde, Fundação Oswaldo Cruz, Fiocruz, Salvador, Brasil.; 6 Secretaria de Vigilância em Saúde e Ambiente, Ministério da Saúde, Brasília, Brasil.; 7 Programa de Pós-graduação em Enfermagem, Universidade Federal de Pelotas, Pelotas, Brasil.

**Keywords:** Hytertension, Diabetes Mellitus, Obesity, Integrality in Health, Family Health, Hipertensión, Diabetes Mellitus, Obesidad, Integralidad en Salud, Salud de la Familia

## Abstract

This study aimed to identify patterns and inequalities in the completeness of care provision for people with hypertension, type 2 diabetes mellitus, and obesity in Brazilian primary health care (PHC), drawing on the 2024 *Brazilian National Basic Health Units Census* (BHU Census). This was a cross-sectional online survey covering 44,938 BHUs registered in the Brazilian National Registry of Health Establishments and validated by municipal health managers. Variables related to geographic region, municipal characteristics, and health service characteristics were analyzed. Care provision was considered complete when the response was positive for all 14 actions related to hypertension, 15 for diabetes, and 10 for obesity. A low proportion of BHUs demonstrated complete provision of care for people with hypertension (15.3%), diabetes (15.6%), and obesity (13.4%), across all regions and municipality profiles. Completeness was higher in the Southeast and Northeast and lower in the South. Large municipalities, municipalities with high or very high social vulnerability, and municipalities with 100% family health coverage also showed higher completeness levels. After adjusting for contextual and BHU characteristics, the family health was associated with 70% higher completeness of care provision. Although access to consultations is well established, weaknesses persist in comprehensiveness and care coordination for chronic diseases, highlighting the need for structural policies that strengthen the capacity of PHC to effectively address health needs.

## Abstract (es)

El estudio tuvo como objetivo establecer patrones y desigualdades en la completitud de la oferta de acciones dirigidas a personas con hipertensión arterial sistémica, diabetes mellitus tipo 2 y obesidad en la atención primaria de salud (APS) brasileña, basándose en el *Censo Nacional de las Unidades Básicas de Salud* (Censo de las UBS), realizado en 2024. Se trata de una encuesta en línea, que abarcó 44.938 UBS registradas en el Catastro Nacional de Establecimientos de Salud y validadas por los gestores municipales. Se analizaron las variables relacionadas con la región geopolítica, el perfil de los municipios y los servicios de salud. Se consideró completa la oferta de atención cuando la respuesta fue positiva para las 14 acciones relacionadas con la hipertensión, las 15 con la diabetes y las 10 con la obesidad. Se observó una baja proporción de UBS con completitud de la oferta de atención a personas con hipertensión (15,3%), diabetes (15,6%) y obesidad (13,4%), en todas las regiones y perfiles de municipio. La completitud de la oferta fue mayor en las regiones Sudeste y Nordeste, y menor en el Sur. Los grandes municipios, así como aquellos con una vulnerabilidad social muy alta y con una cobertura municipal de salud de la familia del 100%, también demostraron una mayor completitud. Tras ajustar por características contextuales y de las UBS, el modelo salud de la familia aumentó en un 70% la completitud de la oferta. Aunque el acceso a consultas está consolidado, siguen existiendo debilidades en la integralidad y en la coordinación de la atención de enfermedades crónicas, lo que exige políticas estructurales que fortalezcan la capacidad resolutiva de la APS.

## Introduction

Comprehensiveness is a foundational principle of the Brazilian Unified National Health System (SUS, acronym in Portuguese), alongside universality and equity, encompassing individual and collective care actions from home-based care and primary health care (PHC) to specialized and hospital services. It also includes health promotion, disease prevention, and surveillance interventions, as well as intersectoral collaboration with areas such as education, labor, and social assistance ^1^.

The operationalization and evaluation of comprehensiveness remain a global challenge, particularly pressing given the increasing prevalence of chronic health conditions ^2^. Noncommunicable diseases (NCDs), such as hypertension, diabetes, and obesity, have become a major global public health challenge in the 21st century, accounting for approximately 75% of all deaths worldwide ^3^. NCDs contribute significantly to premature deaths globally, while also reducing overall quality of life ^4^.

In Brazil, NCDs are the leading causes of death, despite the decline observed between 2000 and 2019 ^5^. It is estimated that by 2030 the country will have a premature mortality rate from NCDs of 274.0 per 100,000 inhabitants, remaining above the Sustainable Development Goals (SDG) target, which calls for a one-third reduction in this indicator by 2030 (225.0 per 100,000 inhabitants) ^6^.

Hypertension affects 24% of the general population and reaches 61% among older adults, while diabetes mellitus has a prevalence of 9% in the general population and 23% among those over 60 years of age ^7^ ^,^ ^8^. Obesity is one of the relevant risk factors for cardiovascular diseases and is directly associated with the occurrence of hypertension and diabetes ^9^ ^,^ ^10^. Data from the Surveillance of Risk and Protective Factors for Chronic Diseases by Telephone Survey (Vigitel, acronym in Portuguese) indicate that excess weight has been increasing, reaching 58.8% in 2021, following the same trend as obesity, which reached 22.1% of Brazilians in the same year ^11^. Findings from the 2013 and 2019 *Brazilian National Health Survey* (PNS, acronym in Portuguese) corroborate the increase in the prevalence of obesity and excess weight in the population, rising from 20.8% to 25.9% and from 57.0% to 60.3%, respectively ^12^. Obesity is associated with increased use of emergency department and basic health units (BHU) services, even after adjusting for sociodemographic variables and sedentary lifestyle ^13^.

NCDs impose a substantial burden on health systems, requiring complex care over time ^3^ ^,^ ^4^. Different models have been adopted around the world to respond to the needs of affected individuals, such as the Chronic Care Model ^14^, widely used to guide care organization by integrating components like clinical decision support, information systems, community linkages, and supported self-management. Their characteristics reflect the health systems in which they are embedded and the resource limitations affecting their full implementation ^15^ ^,^ ^16^.

Within the SUS, the Family Health Strategy (FHS) has dedicated increasing efforts to addressing hypertension, type 2 diabetes mellitus, and obesity. These conditions share modifiable risk factors such as sedentary lifestyle, inadequate diet, and excess weight, but they often coexist, amplifying morbidity, clinical complexity, and health care costs. Implementing comprehensive care for these three chronic conditions requires a comprehensive set of actions in health promotion, disease prevention, treatment, rehabilitation, and palliative care ^17^ ^,^ ^18^.

From 2012 to 2018, the external evaluation of the Improvement Access and Quality National Program of Primary Health Care (PMAQ-AB, acronym in Portuguese) showed advances in NCD care. There was a consistent increase in the ordering of laboratory tests such as serum creatinine measurement, electrocardiography, and lipid profiles, in the provision of specific supplies such as glucometers and blood glucose test strips, and in the scheduling of periodic consultations for the follow-up of people with NCDs ^19^. Even so, only 35% of PHC teams had an organization considered adequate for the care of patients with hypertension and diabetes ^20^.

From the user experience perspective, weaknesses also occurred in comprehensive care, which is fundamental to responding to a complex set of needs and integrating different forms of knowledge and professional practices in the delivery of care. Only 31% of patients with diabetes reported having a foot examination - an essential measure for preventing complications - and less than 20% received all components considered part of comprehensive care ^19^ ^,^ ^20^. Despite institutional and normative advances aimed at their prevention and control, the burden of NCDs remains high, generating significant impacts on the population’s quality of life and on the sustainability of the SUS, on which the majority of Brazilians depend.

Aiming to contribute to addressing the challenges in operationalizing and evaluating comprehensiveness, this study sought to analyze the completeness of care provision for people with hypertension, diabetes, and obesity in PHC and to identify inequalities associated with the municipal context and the characteristics of the BHU, based on the results of the 2024 *Brazilian National Basic Health Units Census* (BHU Census).

## Methods

The 2024 BHU Census is a cross-sectional survey conducted online between June and September 2024. It was led by the Brazilian Ministry of Health, with the objective of producing a detailed diagnosis of the structure and provision of actions in PHC and supporting planning and decision-making in PHC ^21^.

The study population comprised all BHUs registered in the Brazilian National Registry of Health Establishments (CNES, acronym in Portuguese) in May 2024 and validated by municipal health managers. Data collection was carried out using a structured electronic questionnaire completed by the BHU teams, with quality control and technical validation. Details on the methodological development, instrument design, and execution are available in the e-book *Panorama da APS no SUS: Censo Nacional das UBS, 2024* [Overview of PHC in the SUS: Brazilian National BHU Census, 2024; our translation] ^21^ and in Bousquat et al. ^22^ publication.

### Contextual and BHU characteristics

In this study, contextual and BHU characteristics were considered independent variables. This selection was supported by evidence on the relevance and hierarchy of relationships between contextual variables in PHC evaluation studies, in shaping quality standards for comprehensive care for chronic health conditions, and in explaining their differences across the country’s BHUs ^19^ ^,^ ^23^ ^,^ ^24^ ^,^ ^25^.

The most distal contextual characteristics included the major geographic regions of the North, Northeast, Southeast, South, and Central-West and the profile of municipalities, classified according to population size, Social Vulnerability Index (SVI) ^26^, and FHS coverage. Population size categorized municipalities as up to 20,000; from 20,001 to 50,000; from 50,001 to 100,000; from 100,001 to 900,000; and above 900,000 inhabitants. The SVI stratifies municipalities into levels of very high, high, moderate, low, and very low vulnerability. FHS coverage for 2023 expresses the percentage of the municipality’s population covered by teams of this care model, grouped into four categories: below 50%, between 50% and 74.9%, between 75% and 99.9%, and 100% ^27^.

The BHUs were characterized with respect to the care model, physical infrastructure, team composition, technological infrastructure, and organizational aspects. The care model identified the presence of at least one FHS team in the BHU (yes, no). The physical infrastructure assessed the availability of eight items essential for the care of people with hypertension, diabetes, or obesity: (i) adult stethoscope, (ii) adult sphygmomanometer, (iii) large adult blood pressure cuff for people with obesity, (iv) glucometer, (v) blood glucose test strips, (vi) measuring tape, (vii) digital oximeter, and (viii) mechanical or digital scale with a capacity of up to 200kg (yes, no).

The composition of the multidisciplinary team at each BHU included the presence of four or more community health workers (CHW) (yes, no), of professionals from multidisciplinary teams (yes, no), and of physicians and nurses with specialization in Family and Community Medicine (FCM) or Family Health (yes, no). The technological infrastructure was examined through the BHU’s adequate internet connectivity (yes, no) and the use of electronic health records (yes, no).

The organizational aspects of service provision by the BHU were characterized by team participation in continuing education activities (always or almost always, sometimes or never), the use of vulnerability and risk criteria for population empanelment (yes, no), and the existence of an active local health council (yes, no).

### Care provision for people with hypertension, diabetes, and obesity in PHC

To operationalize the concept of comprehensive care ^1^ ^,^ ^25^, the three study outcomes were characterized by the completeness of care provision in the BHUs. A set of health actions, both common and specific to each condition in this triad of chronic conditions, was considered, including consultations, home visits, educational groups, condition-specific patient registries, body practices and physical activity, counseling, monitoring of clinical parameters, risk stratification, and ordering of diagnostic tests. Care provision for each condition was considered complete when the response was positive for all evaluated actions. For hypertension, there were 14 actions; for diabetes, 15 actions; and for obesity, 10 actions (Box 1), available in the e-book ^21^ for Brazil and its major regions.

The selection of health actions took into account clinical and programmatic relevance in addressing NCDs in PHC, based on official documents ^28^ ^,^ ^29^ ^,^ ^30^ ^,^ ^31^ ^,^ ^32^, protocols, and evidence from studies that deepened the understanding of the characterization of comprehensive and effective care for the population and patients of the country’s BHUs ^19^ ^,^ ^23^ ^,^ ^24^ ^,^ ^25^ ^,^ ^31^ ^,^ ^32^. All variables available in the instrument for each condition were used for the completeness analysis. In the instrument development process, the scope of actions was defined by the technical departments of the Brazilian Ministry of Health, grounded in protocols and official documents.

**Box 1:** Selected actions for analysis of completeness of care for people with hypertension, diabetes, and obesity. 2024 *Brazilian National Basic Health Units Census*.

	HYPERTENSION	DIABETES	OBESITY
Medical consultation for follow-up			
Nursing consultation as part of continuous care			
CHW home visit for active outreach to high-risk patients or those who missed appointments			
Collective activities in educational groups as part of continuous care			
Updated registry of people with the condition for follow-up			
Updated registry of patients referred to other points of the health care network for follow-up			
Body practices and physical activities promoted in the BHU or in the catchment area			
Provision of counseling on adequate and healthy eating, based on the *Dietary Guidelines for the Brazilian Population*			
Blood pressure measurement and recording as part of routine follow-up and hypertension control			
Nutritional status assessment (weight and height) with recording in the clinical record			
Dietary intake monitoring with food consumption markers in the electronic health record			
Cardiovascular risk stratification with recording in the clinical record			
Ordering of serum creatinine with recording in the clinical record			
Ordering of lipid profile with recording in the clinical record			
Ordering of ECG with recording in the clinical record			
Ordering of fasting blood glucose with recording in the clinical record			
Ordering of glycated hemoglobin with recording in the clinical record			
Foot examination as part of continuous care			
Counseling on foot self-care as part of continuous care			
Counseling on insulin use as part of continuous care			

BHU: basic health units; CHW: community health workers.

Source: prepared by the authors based on information published in the e-book *Panorama da APS no SUS: Censo Nacional das UBS, 2024* [Overview of PHC in the SUS: Brazilian National BHU Census, 2024; our translation] ^21^.

### Theoretical model, categories, and analysis procedures

Descriptive analyses were performed to estimate the absolute and relative frequencies of each action composing the characterization of completeness of care provision, as well as completeness itself, for each condition - hypertension, diabetes, and obesity - in the country’s BHUs.

The multivariate analysis used a multilevel hierarchical approach ^23^ ^,^ ^24^ ^,^ ^33^ to adjust the effect of context and BHU characteristics on the completeness of care provision for the three chronic conditions. This approach recognizes the direct dependence of BHUs (level 1) on the municipal contexts (level 2) in which they are located, expressed as an intraclass correlation between PHC services within the same municipality. This modeling approach was necessary given the complex structure and asymmetry of the data, where small municipalities often have a single BHU, while large urban centers have dozens or hundreds of BHUs. By accounting for the intraclass correlation (ICC) resulting from this contextual nesting, the approach corrects for data dependence and ensures more accurate and valid statistical estimates about care provision. At level 1, the results for each variable were adjusted for all BHU variables (level 1) and all context variables (level 2). At level 2, the results for each variable were adjusted for all contextual variables.

The multilevel model used Poisson regression with robust variance, estimating crude and adjusted prevalence ratios (PR) and respective 95% confidence intervals (95%CI). The p-value was calculated using the Wald test for heterogeneity for categorical variables and a linear trend test for continuous variables, considering a p-value < 0.05 as significant. All analyses were performed in Stata, version 17.0 (https://www.stata.com).

### Ethical aspects

The research protocol was reviewed and approved by the Research Ethics Committee of the School of Public Health, University of São Paulo (approval number 6,779,222, issued on April 25, 2024, CAAE: 78767024.2.0000.5421).

## Results

### Distribution of BHUs according to contextual factors

The 2024 BHU Census included 44,938 BHUs across the country. The highest concentration of BHUs was found in the Northeast (39.5%), followed by the Southeast (29.7%) and the South (14.7%), while the North (9.1%) and Central-West (7%) accounted for the smallest proportions ^21^ ^,^ ^22^.

Regarding population size, 34.6% of BHUs were located in municipalities with up to 20,000 inhabitants, 25.4% in municipalities from 20,001 to 50,000, 13.4% in municipalities from 50,001 to 100,000, 21.5% in municipalities from 100,001 to 900,000, and 5.2% in municipalities above 900,000. The distribution of BHUs by SVI revealed that more than one-third were in municipalities with high (21.4%) or very high vulnerability (15.1%), 23.5% with moderate vulnerability, 29.7% with low vulnerability, and 10.2% with very low social vulnerability. The distribution of BHUs according to municipal FHS coverage indicates that the majority (59.3%) were in municipalities with 100% coverage, 17.9% in municipalities between 75% and 99.9%, and 14.4% in municipalities with coverage between 50% and 74.9% ^21^.

### Distribution of BHUs according to service characteristics

In 88.5% of BHUs there was at least one FHS team, and 32.4% had all eight items of essential equipment and supplies. Regarding the BHU team composition, 74.7% reported the presence of four or more CHWs, 42.2% received support from multidisciplinary teams, and 17.3% had physicians and nurses with specialization in FCM or Family Health. Adequate internet connectivity was reported by 65.2%, and the use of electronic health records was reported by 87.3% of BHUs. Regarding organizational characteristics, 64.8% confirmed that the team participated in continuing education activities always or almost always, 59.9% used vulnerability and risk criteria for population empanelment, and 36.3% had an active local health council ^21^.

### Provision of health actions for people with hypertension, diabetes, and obesity

Actions provided by more than 80% of BHUs were medical and nursing consultations for all conditions, CHW home visits for hypertension and diabetes; blood pressure measurement and recording and the ordering of serum creatinine, lipid profile, and electrocardiogram (ECG) tests for hypertension; ordering of fasting blood glucose and glycated hemoglobin for diabetes; and weight and height assessment for obesity (Table 1).

**Table 1:** Proportion of Brazilian Unified National Health System (SUS, acronym in Portuguese) basic health units (BHU) providing health actions in the care of people with hypertension, diabetes, and obesity. 2024 *Brazilian National Basic Health Units Census*, (N = 44,938).

Health actions *	Hypertension		Diabetes		Obesity	
	n	%	n	%	n	%
Medical consultation	43,828	97.5	43,881	97.7	41,733	92.9
Nursing consultation	42,136	93.8	42,252	94.0	37,845	84.2
CHW home visit	38,323	85.3	37,661	83.8	31,124	69.3
Collective activities in educational groups	30,114	67.0	28,671	63.8	19,728	43.9
Updated registry of people with the condition	34,227	76.2	33,985	75.6	16,670	37.1
Updated registry of people with the condition referred to other points of the health care network	19,589	43.6	19,820	44.1	12,365	27.5
Body practices and physical activities	19,300	43.0	19,258	42.9	17,536	39.0
Counseling on adequate and healthy eating	35,088	78.1	33,110	73.7	25,948	57.8
Blood pressure measurement and recording	43,699	97.3	-	-	-	-
Nutritional status assessment (weight and height)	-	-	31,150	69.3	38,522	85.7
Dietary intake monitoring	22,328	49.7	-	-	24,894	55.4
Cardiovascular risk stratification	27,539	61.3	29,011	64.6	-	-
Ordering of serum creatinine test	39,221	87.3	-	-	-	-
Ordering of lipid profile test	37,069	82.5	-	-	-	-
Ordering of ECG test	38,807	86.4	-	-	-	-
Ordering of fasting blood glucose	-	-	42,577	94.8	-	-
Ordering of glycated hemoglobin	-	-	42,528	94.6	-	-
Foot examination	-	-	26,354	58.7	-	-
Counseling on foot self-care	-	-	35,019	77.9	-	-
Counseling on insulin use	-	-	38,743	86.2	-	-

CHW: community health workers.

* Information published in the e-book *Panorama da APS no SUS: Censo Nacional das UBS, 2024* [Overview of PHC in the SUS: Brazilian National BHU Census, 2024; our translation] ^21^.

Prevalences between 51% and 79% among BHUs were observed for counseling on adequate and healthy eating for all conditions; collective activities in educational groups and an updated patient registry for the condition; cardiovascular risk stratification; weight and height assessment for hypertension and diabetes; foot examination and counseling on foot self-care for people with diabetes; and CHW home visits and dietary intake monitoring for obesity (Table 1).

Prevalences between 27% and 50% among BHUs were recorded for maintaining an updated registry of patients with the condition referred to other services within the health care network; body practices and physical activity for all conditions; dietary intake monitoring for hypertension; and collective activities in educational groups and an updated patient registry for obesity (Table 1).

### Completeness of care provision for people with hypertension, diabetes, and obesity

Tables 2, 3 and 4 (hypertension, diabetes and obesity, respectively) present the distribution of completeness of care provision and the associations with contextual and BHU characteristics, through multivariate, crude, and adjusted analyses. In Brazil, 15.3% (n = 6,863) of BHUs offered complete provision of care to people with hypertension, 15.6% (n = 7,008) to those with diabetes, and 13.4% (n = 6,018) to those with obesity.

**Table 2:** Completeness of care provision for people with hypertension, by region, municipality profile, and basic health units (BHU) characteristics. 2024 *Brazilian National Basic Health Units Census* (N = 44,938).

	Completeness frequency		Crude		Adjusted *	
	n	%	PR	95%CI	PR	95%CI
Context characteristics (level 2)						
Region				p < 0.001		p = 0.001
Central-West	412	13.2	1.36	1.12-1.65	1.23	1.04-1.46
Northeast	2,900	16.4	1.74	1.52-1.99	1.19	1.01-1.40
North	544	13.3	1.36	1.13-1.63	1.06	0.87-1.28
Southeast	2,376	17.8	1.67	1.45-1.92	1.52	1.34-1.72
South	631	9.6	Reference		Reference	
Population size (inhabitants)				p = 0.004		p = 0.001
Above 900,000	658	28.0	1.66	1.12-2.44	1.91	1.36-2.68
From 100,001 to 900,000	1,310	13.6	0.91	0.79-1.05	1.18	1.01-1.39
From 50,001 to 100,000	877	14.6	0.92	0.80-1.07	1.09	0.95-1.26
From 20,001 to 50,000	1,596	14.0	0.87	0.79-0.97	0.95	0.86-1.04
Up to 20,000	2,422	15.6	Reference		Reference	
Social Vulnerability Index				p < 0.001		p = 0.001
Very high	1,081	16.0	1.47	1.23-1.75	1.37	1.12-1.68
High	1,651	17.1	1.61	1.36-1.90	1.37	1.13-1.64
Moderate	1,570	14.9	1.35	1.14-1.59	1.14	0.97-1.34
Low	2,038	15.3	1.18	1.00-1.39	1.01	0.87-1.17
Very low	523	11.4	Reference		Reference	
Family Health coverage (%)				p < 0.001		p < 0.001
Below 50	463	12.2	Reference		Reference	
Between 50 and 74.9	931	14.4	1.73	1.31-2.28	1.55	1.21-2.00
Between 75 and 99.9	1,109	13.8	1.99	1.54-2.58	1.99	1.55-2.56
100	4,360	16.4	2.65	2.08-3.36	2.40	1.85-3.13
BHU characteristics (level 1)						
Care model				p < 0.001		p < 0.001
FHS	6,564	16.5	2.90	2.31-3.62	1.69	1.36-2.11
Other	287	5.6	Reference		Reference	
Equipment and supplies (8 items) **				p < 0.001		p < 0.001
Yes	2,823	19.4	1.34	1.28-1.41	1.25	1.19-1.31
No	4,039	13.3	Reference		Reference	
BHU with 4 or more CHWs				p < 0.001		p < 0.001
Yes	5,876	17.5	1.87	1.65-2.10	1.26	1.16-1.38
No	985	8.7	Reference		Reference	
BHU with multidisciplinary teams				p < 0.001		p < 0.001
Yes	3,940	20.8	1.58	1.48-1.68	1.31	1.24-1.39
No	2,923	11.3	Reference		Reference	
Physician and nurse with specialization in FCM/Family Health				p < 0.001		p < 0.001
Yes	1,575	20.3	1.33	1.26-1.41	1.18	1.11-1.25
No	5,286	14.2	Reference		Reference	
Adequate internet connectivity in the BHU				p < 0.001		p = 0.010
Yes	4,925	16.8	1.21	1.14-1.28	1.07	1.02-1.13
No	1,937	12.4	Reference		Reference	
BHU uses electronic health record				p < 0.001		p < 0.001
Yes	6,361	16.2	1.81	1.63-2.02	1.38	1.23-1.53
No	501	8.8	Reference		Reference	
Professionals participate in continuing education				p < 0.001		p < 0.001
Always or almost always	5,746	19.7	2.34	2.18-2.52	1.94	1.81-2.09
Sometimes or never	1,116	7.1	Reference		Reference	
BHU uses vulnerability and risk criteria for population empanelment				p < 0.001		p < 0.001
Yes	5,500	20.4	2.27	2.10-2.46	1.87	1.72-2.04
No	1,361	7.6	Reference		Reference	
Active local health council				p < 0.001		p < 0.001
Yes	3,553	21.8	1.65	1.56-1.74	1.41	1.33-1.49
No	3,308	11.6	Reference		Reference	
Total	6,863	15.3				

95%CI: 95% confidence interval; CHW: community health workers; FCM: Family and Community Medicine; FSH: Family Health Strategy; PR: prevalence ratio.

* * At level 1, the results for each variable were adjusted for all BHU characteristic variables and all level 2 variables (context characteristics). At level 2, the results for each variable were adjusted for all context characteristic variables;

** Eight items essential for the care of people with hypertension, diabetes, or obesity: (i) adult stethoscope, (ii) adult sphygmomanometer, (iii) large adult blood pressure cuff for people with obesity, (iv) glucometer, (v) blood glucose test strips, (vi) measuring tape, (vii) digital oximeter, and (viii) mechanical or digital scale with a capacity of up to 200kg.

**Table 3:** Completeness of care provision for people with diabetes, by region, municipality profile, and primary health care unit characteristics. 2024 *Brazilian National Basic Health Units Census* (N = 44,938).

	Completeness frequency		Crude		Adjusted *	
	n	%	PR	95%CI	PR	95%CI
Context characteristics (level 2)						
Region				p < 0.001		p = 0.001
Central-West	383	12.3	1.11	0.91-1.34	1.02	0.86-1.22
Northeast	2,953	16.7	1.55	1.36-1.77	1.11	0.95-1.30
North	521	12.7	1.08	0.90-1.31	0.87	0.72-1.06
Southeast	2,442	18.3	1.45	1.26-1.66	1.33	1.18-1.51
South	709	10.7	Reference		Reference	
Population size (inhabitants)				p = 0.008		p = 0.001
Above 900,000	725	30.9	1.86	1.25-2.77	2.15	1.52-3.05
From 100,001 to 900,000	1,425	14.8	1.03	0.90-1.18	1.34	1.14-1.57
From 50,001 to 100,000	845	14.1	0.91	0.78-1.05	1.08	0.94-1.24
From 20,001 to 50,000	1,662	14.6	0.93	0.84-1.04	1.02	0.92-1.12
Up to 20,000	2,351	15.1	Reference		Reference	
Social Vulnerability Index				p < 0.001		p = 0.006
Very high	1,071	15.8	1.32	1.11-1.57	1.25	1.02-1.52
High	1,611	16.7	1.40	1.19-1.65	1.22	1.01-1.46
Moderate	1,627	15.4	1.23	1.04-1.45	1.08	0.92-1.27
Low	2,116	15.8	1.06	0.90-1.25	0.94	0.82-1.09
Very low	583	12.7	Reference		Reference	
Family Health coverage (%)				p < 0.001		p = 0.001
Below 50	533	14.1	Reference		Reference	
Between 50 and 74.9	994	15.4	1.70	1.29-2.24	1.52	1.18-1.95
Between 75 and 99.9	1,143	14.2	1.83	1.41-2.36	1.86	1.46-2.37
100	4,338	16.3	2.41	1.90-3.06	2.32	1.80-2.98
BHU charatcteristics (level 1)						
Care model				p < 0.001		p < 0.001
FHS	6,703	16.9	2.90	2.32-3.64	1.71	1.38-2.10
Other	295	5.7	Reference		Reference	
Equipment and supplies (8 items) **				p < 0.001		p < 0.001
Yes	2,938	20.2	1.36	1.29-1.43	1.26	1.20-1.33
No	4,069	13.4	Reference		Reference	
BHU with 4 or more CHWs				p < 0.001		p < 0.001
Yes	6,033	18.0	1.94	1.71-2.20	1.35	1.23-1.47
No	974	8.6	Reference		Reference	
BHU with multidisciplinary teams				p < 0.001		p < 0.001
Yes	4,046	21.3	1.60	1.51-1.70	1.35	1.27-1.42
No	2,962	11.4	Reference		Reference	
Physician and nurse with specialization in FCM/Family Health				p < 0.001		p < 0.001
Yes	1,620	20.8	1.30	1.22-1.37	1.14	1.08-1.21
No	5,385	14.5	Reference		Reference	
Adequate internet connectivity in the BHU				p < 0.001		p < 0.001
Yes	5,042	17.2	1.22	1.16-1.29	1.09	1.04-1.15
No	1,964	12.5	Reference		Reference	
BHU uses electronic health record				p < 0.001		p < 0.001
Yes	6,429	16.4	1.59	1.44-1.76	1.17	1.06-1.30
No	577	10.1	Reference		Reference	
Professionals participate in continuing education				p < 0.001		p < 0.001
Always or almost always	5,864	20.1	2.26	2.11-2.43	1.89	1.77-2.03
Sometimes or never	1,143	7.2	Reference		Reference	
BHU uses vulnerability and risk criteria for population empanelment				p < 0.001		p < 0.001
Yes	5,552	20.6	2.16	2.00-2.33	1.79	1.66-1.94
No	1,454	8.1	Reference		Reference	
Active local health council				p < 0.001		p < 0.001
Yes	3,600	22.1	1.58	1.49-1.67	1.36	1.29-1.44
No	3,406	11.9	Reference		Reference	
Total	7,008	15.6				

95%CI: 95% confidence interval; CHW: community health workers; FCM: Family and Community Medicine; FSH: Family Health Strategy; PR: prevalence ratio.

* * At level 1, the results for each variable were adjusted for all BHU characteristic variables and all level 2 variables (context characteristics). At level 2, the results for each variable were adjusted for all context characteristic variables;

** Eight items essential for the care of people with hypertension, diabetes, or obesity: (i) adult stethoscope, (ii) adult sphygmomanometer, (iii) large adult blood pressure cuff for people with obesity, (iv) glucometer, (v) blood glucose test strips, (vi) measuring tape, (vii) digital oximeter, and (viii) mechanical or digital scale with a capacity of up to 200kg.

**Table 4:** Completeness of care provision for people with obesity, by region, municipality profile, and basic health units (BHU) characteristics. 2024 *Brazilian National Basic Health Units Census* (N = 44,938).

	Completeness frequency		Crude		Adjusted *	
	n	%	PR	95%CI	PR	95%CI
Context characteristics (level 2)						
Region				p < 0.001		p = 0.001
Central-West	340	10.9	1.39	1.13-1.72	1.27	1.05-1.54
Northeast	2,705	15.3	2.02	1.75-2.34	1.47	1.23-1.75
North	482	11.8	1.48	1.21-1.81	1.22	0.99-1.51
Southeast	1,993	14.9	1.70	1.46-1.99	1.58	1.37-1.81
South	498	7.5	Reference		Reference	
Population size (inhabitants)				p = 0.014		p = 0.951
Above 900,000	541	23.0	1.18	0.65-2.11	1.17	0.68-2.02
From 100,001 to 900,000	1,044	10.8	0.80	0.69-0.93	0.99	0.83-1.17
From 50,001 to 100,000	725	12.1	0.82	0.70-0.97	0.96	0.83-1.12
From 20,001 to 50,000	2,191	14.1	0.92	0.82-1.03	1.00	0.91-1.11
Up to 20,000	1,517	13.3	Reference		Reference	
Social Vulnerability Index				p < 0.001		p = 0.037
Very high	980	14.5	1.61	1.33-1.95	1.20	0.96-1.50
High	1,542	16.0	1.79	1.49-2.14	1.25	1.02-1.54
Moderate	1,397	13.2	1.44	1.20-1.74	1.13	0.94-1.35
Low	1,685	12.6	1.18	0.98-1.42	0.98	0.83-1.15
Very low	414	9.0	Reference		Reference	
Family Health coverage (%)				p < 0.001		p < 0.001
Below 50	389	10.3	Reference		Reference	
Between 50 and 74.9	792	12.2	1.73	1.22-2.44	1.71	1.29-2.27
Between 75 and 99.9	887	11.1	1.87	1.36-2.56	1.85	1.40-2.44
100	3,950	14.8	2.77	2.07-3.71	2.30	1.72-3.06
BHU characteristics (level 1)						
Care model				p < 0.001		p < 0.001
FHS	5,764	14.5	2.98	2.40-3.69	1.75	1.42-2.16
Other	241	4.7	Reference		Reference	
Equipment and supplies (8 items) **				p < 0.001		p < 0.001
Yes	2,462	16.7	1.37	1.29-1.45	1.28	1.21-1.36
No	3,554	11.7	Reference		Reference	
BHU with 4 or more CHWs				p < 0.001		p < 0.001
Yes	5,127	15.3	1.81	1.59-2.05	1.20	1.10-1.31
No	889	7.8	Reference		Reference	
BHU with multidisciplinary teams				p < 0.001		p < 0.001
Yes	3,533	18.6	1.69	1.59-1.80	1.40	1.32-1.48
No	2,485	9.6	Reference		Reference	
Physician and nurse with specialization in FCM/Family Health				p < 0.001		p < 0.001
Yes	1,365	17.6	1.36	1.28-1.44	1.20	1.13-1.28
No	4,651	12.5	Reference		Reference	
Adequate internet connectivity in the BHU				p < 0.001		p = 0.004
Yes	4,356	14.9	1.23	1.16-1.31	1.09	1.03-1.16
No	1,661	10.6	Reference		Reference	
BHU uses electronic health record				p < 0.001		p < 0.001
Yes	5,542	14.1	1.67	1.49-1.87	1.27	1.13-1.42
No	475	8.3	Reference		Reference	
Professionals participate in continuing education				p < 0.001		p < 0.001
Always or almost always	5,113	17.6	2.58	2.38-2.80	2.11	1.95-2.28
Sometimes or never	904	5.7	Reference		Reference	
BHU uses vulnerability and risk criteria for population empanelment				p < 0.001		p < 0.001
Yes	4,872	18.1	2.41	2.20-2.64	1.96	1.79-2.15
No	1,144	6.4	Reference		Reference	
Active local health council				p < 0.001		p < 0.001
Yes	3,216	19.7	1.82	1.71-1.94	1.55	1.46-1.65
No	2,800	9.8	Reference		Reference	
Total	6,018	13.4				

95%CI: 95% confidence interval; CHW: community health workers; FCM: Family and Community Medicine; FSH: Family Health Strategy; PR: prevalence ratio.

* * At level 1, the results for each variable were adjusted for all BHU characteristic variables and all level 2 variables (context characteristics). At level 2, the results for each variable were adjusted for all context characteristic variables;

** Eight items essential for the care of people with hypertension, diabetes, or obesity: (i) adult stethoscope, (ii) adult sphygmomanometer, (iii) large adult blood pressure cuff for people with obesity, (iv) glucometer, (v) blood glucose test strips, (vi) measuring tape, (vii) digital oximeter, and (viii) mechanical or digital scale with a capacity of up to 200kg.

### Completeness according to contextual characteristics

In the crude multilevel analysis, BHUs in the Northeast showed superior performance regarding completeness of care provision. Compared to the South region, Northeastern BHUs exhibited a higher probability of complete provision of care for hypertension (PR = 1.74; 95%CI: 1.52-1.99), diabetes (PR = 1.55; 95%CI: 1.36-1.77), and obesity (PR = 2.00; 95%CI: 1.75-2.34). BHUs in the Southeast also stood out, with completeness levels 45% to 70% higher than those observed in the South. In turn, the Central-West and North showed significantly higher levels than the South for hypertension and obesity, while for diabetes the results were similar across these regions.

After adjusting for contextual variables, regional inequalities were attenuated. In the adjusted analysis, BHUs in the Southeast showed the highest completeness levels, 50% higher than in the South. Although the advantage of the Northeast diminished, the region maintained significant differences for hypertension and obesity and a pattern similar to the South for diabetes. BHUs in the Central-West showed a pattern similar to the Northeast, while those in the North no longer differed significantly from those in the South.

In the crude analysis, BHUs in municipalities with more than 900,000 inhabitants presented completeness levels for hypertension and diabetes significantly higher than those in other population sizes. After adjusting for contextual variables, BHUs in the largest municipalities had a probability of completeness for hypertension and diabetes that was significantly higher than that of BHUs in medium-sized and small municipalities. For obesity, completeness was similar across population sizes, both in crude and adjusted analyses.

BHUs in municipalities with high or very high social vulnerability presented, in the crude analysis, completeness levels 50% to 60% higher for hypertension, 30% to 40% higher for diabetes, and 60% to 80% higher for obesity when compared to those in municipalities with lower vulnerability. After adjusting for contextual variables, BHUs in more vulnerable municipalities maintained significantly higher completeness - between 20% and 40% - for the three conditions analyzed. BHUs in municipalities with low or very low SVI performed similarly to each other but consistently lower than those in more vulnerable contexts.

A positive association was also observed between FHS coverage and completeness of care provision for this triad of chronic conditions. This association persisted after adjusting for contextual variables: BHUs located in municipalities with 100% FHS coverage were twice as likely to achieve completeness compared to those in municipalities with less than 50% coverage.

### Completeness according to BHU characteristics

In the crude analysis, BHUs operating under the FHS care model had an almost three-fold higher probability of complete provision of care for hypertension, diabetes, and obesity compared to BHUs without FHS. After adjusting for context and BHU variables, the effect of the care model was reduced but remained significant, with an approximately 70% higher probability in BHUs with FHS teams.

The availability of the eight essential pieces of equipment and supplies was also positively associated with completeness of care. In the crude analysis, these BHUs showed about 35% higher completeness for the three conditions. The association remained significant after adjustment: units with all essential equipment and supplies had about a 25% higher probability than those without.

Regarding team composition, BHUs with four or more CHWs had, in the crude analysis, completeness levels 80% to 95% higher than those with fewer CHWs. After adjustment, the magnitude decreased but remained statistically significant, with completeness levels 20% to 30% higher in BHUs with four or more CHWs.

Similarly, the presence of physicians and nurses with specialization in Family and Community Medicine or Family Health was associated with better results. In the crude analysis, completeness levels were 30% to 40% higher in these PHCUs, and after adjustment they remained 15% to 20% higher compared to those without professionals with this training.

BHUs receiving support from multidisciplinary teams had, in the crude analysis, completeness levels 60% to 70% higher than those without such support. Even after adjustment, the association remained significant: units with multidisciplinary teams support showed 30% to 40% higher completeness levels in hypertension, diabetes, and obesity care.

Internet connectivity was also relevant in the crude analysis: BHUs with adequate connectivity had approximately 20% higher completeness of care than those with poor access. After adjustment, completeness was about 10% higher in BHUs with adequate internet access. The use of electronic health records was associated with 60% to 80% higher completeness of care in the crude analysis; after adjustment, BHUs with this tool had 20% to 40% higher completeness of care than those without it.

Team qualification also showed a robust effect. BHUs whose professionals always or almost always participated in continuing education activities had, in the crude analysis, completeness levels 2.0 to 2.6 times higher than those of BHUs with occasional or no participation. After adjustment, the association remained significant, with completeness levels 90% to 110% higher in BHUs with teams regularly engaged in continuing education activities.

Another highlight was the use of vulnerability and risk criteria for population empanelment, which in the crude analysis showed a probability of completeness of care 2.0 to 2.4 times higher than in BHUs that did not use such criteria. The difference remained significant after adjustment, with an 80% to 96% higher probability of completeness of care in BHUs that adopted vulnerability criteria.

Finally, the presence of an active local health council was associated, in the crude analysis, with completeness levels 60% to 80% higher than in units without this structure. After adjustment, the association persisted, with a 35% to 55% higher probability of completeness of care in BHUs with a functioning local health council.

The estimated ICC in the multilevel analysis was ≥ 0.80, indicating a strong dependence of BHUs on municipal contextual characteristics in shaping the completeness of care provision for the three conditions ^33^.

## Discussion

The results of this study indicate that approximately 85% of SUS BHUs did not achieve complete provision of care for people with hypertension, diabetes, and obesity. They also reveal significant variability in this proxy indicator of comprehensive care, depending on contextual characteristics and the PHC services themselves.

Basic clinical actions, such as consultations and measurement of clinical parameters, are widely institutionalized in the routine of BHUs for the three conditions studied. CHW home visits for active outreach to patients who missed scheduled consultations were widely reported for hypertension and diabetes, similar to findings from the first cycle of PMAQ ^19^, indicating the historical priority of these conditions in PHC, while the lower percentage related to obesity suggests that obesity is less integrated into routine care strategies.

Nutritional status assessment was more modest for diabetes, suggesting weaknesses in the regular monitoring of essential clinical parameters for people at metabolic risk. There was also low incorporation of educational, preventive, and interprofessional practices. Obesity, in particular, was associated with lower proportions of educational practices and counseling based on the *Dietary Guidelines for the Brazilian Population* ^34^ ^,^ ^35^.

The small number of BHUs that maintained patient follow-up registries indicates gaps in longitudinal surveillance and care coordination, especially for obesity. The limited provision of these actions suggests fragmented care for the three conditions and the potential underutilization of preventive strategies for problems that share risk factors and frequently co-occur, configuring one of the most common multimorbidity profiles ^36^ ^,^ ^37^ ^,^ ^38^.

Inequalities in the completeness of care provision for hypertension, diabetes, and obesity reflect both contextual and service characteristics. BHUs in the Southeast and Northeast stood out with the highest completeness levels. The Southeast also includes more populous municipalities, with more developed health care infrastructure, while the Northeast encompasses municipalities with a higher proportion of BHUs operating under the FHS care model. Thus, well-equipped and well-organized BHUs with complete FHS teams and community-oriented actions were associated with significantly higher proportions of comprehensive care provision in the care of people with hypertension, diabetes, and obesity. This finding is consistent with the higher completeness of actions for hypertension and diabetes ^20^ ^,^ ^38^ ^,^ ^39^ in large municipalities, especially those with more than 900,000 inhabitants, and in municipalities with 100% population coverage by the FHS.

PHC financing has historically benefited municipalities with greater vulnerability ^40^, and there is evidence of more equitable results in PHC evaluations, which corroborates the results of PHCUs in more vulnerable municipalities ^41^. In the Northeast, the better performance associated with higher FHS coverage has contributed to a substantial reduction in long-standing inequalities, already noticeable in cycle three of PMAQ ^20^ ^,^ ^38^ ^,^ ^39^ ^,^ ^41^ ^,^ ^42^. The expansion of municipal FHS coverage showed a strong association with increased completeness of provision. The South and the North, for different reasons, showed the worst situations. The North is the region with the most limited health infrastructure, the greatest access challenges, and the greatest difficulties in attracting and retaining health professionals. The South appears to have lost its prominence in PHC over time, with its primary care network no longer ranking among the best in the country, a situation already observed in 2018, in cycle three of PMAQ ^20^ ^,^ ^38^ ^,^ ^39^ ^,^ ^41^ ^,^ ^43^. It is important to note that data collection occurred in 2024, a period in which Rio Grande do Sul was severely affected by an extreme weather event that directly and indirectly impaired the infrastructure of BHUs and their capacity to provide care. However, even after excluding the BHUs from Rio Grande do Sul from the analysis, the South region remained in last place.

The higher completeness of provision in BHUs with at least one FHS team is also consistent with studies conducted over the past two decades in different regions of the country ^23^ ^,^ ^24^. The effect of the FHS is also expressed in the association between completeness and the presence of four or more CHWs, the use of vulnerability and risk criteria for population empanelment, and the existence of an active local health council. The relevance of the FHS care model in improving care quality has been associated with territorial empanelment, community engagement and social participation, longitudinal follow-up of patients, and the implementation of health promotion, prevention, and control actions for chronic health conditions ^44^. Thus, the results indicate the FHS’s capacity to coordinate and manage individual and community interventions in a comprehensive and equitable manner through active outreach, systematic and continuous follow-up of people with chronic conditions, use of clinical protocols, and coordination with other levels of care ^45^ ^,^ ^46^. Several studies show that higher municipal FHS coverage is associated with better health outcomes, such as reduced hospitalizations for PHC-sensitive conditions ^47^ ^,^ ^48^.

The completeness of care provision was positively associated with support from multidisciplinary teams, a result consistent with the literature highlighting the importance of an interdisciplinary approach for the effective management of chronic conditions ^49^ ^,^ ^50^. The positive performance of BHUs with multidisciplinary teams support can be explained by the expansion of matrix support and by the strengthening of collaborative work among teams, fundamental aspects for comprehensive and longitudinal care, reinforcing PHC’s role as care coordinator and organizer of the health care network. Matrix support goes beyond periodic meetings and involves continuing education activities, technical supervision, and shared care, integrating knowledge and practices and thus providing teams with additional resources and expertise to manage complex situations ^51^ ^,^ ^52^. However, it is important to recognize that the availability of multidisciplinary teams in PHC is still insufficient to meet the growing burden of chronic conditions.

BHUs with professionals engaged in continuing education activities and those with professionals specialized in FCM and family health nursing showed higher completeness of actions. The literature indicates that continuing education activities, by fostering critical reflection on the work process and continuous technical updating, contributes to strengthening clinical practices, expanding the capacity to address health needs, and incorporating approaches centered on supported self-management and risk management ^53^. Successful experiences show that the consolidation of continuing education activities in the routine of BHUs results in greater patient engagement in care, fewer missed consultations, and better care coordination ^54^ ^,^ ^55^.

International evidence indicates that comprehensive PHC, organized longitudinally by multidisciplinary teams that work collaboratively and are regularly trained, is associated with greater benefits for chronic disease care and patient satisfaction. Barriers to comprehensive care are similar to those observed in this study, with emphasis on inadequate service infrastructure, work overload among teams, fragmented information systems, scarcity of diagnostic and therapeutic resources, and bottlenecks and long waiting times for access to specialists ^56^ ^,^ ^57^ ^,^ ^58^.

A strength of our approach was the selection of variables used to compose the completeness indicator. The choice was guided by criteria based on current guidelines, seeking to represent recommended actions. The construct of completeness of care provision revealed the persistent challenges in ensuring comprehensive care for people with hypertension, diabetes, and obesity in PHC. However, it also has limitations, as some actions important for the comprehensive care of the three conditions were assessed for only one of them - for example, blood pressure measurement was considered only for people with hypertension, and not for those with diabetes or obesity.

This study includes exclusively data from the 2024 BHU Census and therefore shares its strengths and limitations. As an online census survey, this study is subject to the limitations inherent to this type of design. We highlight the possibility of information bias, as respondents were indicated by managers, which may have led to inaccuracies in the responses. However, we observed no limitations related to the study design, the inclusion of BHUs, or the analyses. Moreover, it is important to emphasize the multilevel modeling adopted, which considers the hierarchical structure of the data, producing more robust estimates and, after adjustment, highlighting the contribution of each context and BHU characteristic to completeness of care provision. The ICC ≥ 0.80 indicates a strong dependence of BHUs on the municipal context, whose characteristics shape the observed inequalities in completeness of care provision.

This study highlights the importance of further investigating this triad of chronic conditions, given the growth of NCDs and their frequent co-occurrence. Among these conditions, the completeness of care provision for obesity stands out as a systemic challenge. Future studies aimed at triangulating information, integrating new databases, and deepening the analysis of the quality and effectiveness of PHC actions may improve monitoring and support more responsive public policies. The continuity of evaluation processes is essential, especially when combined with in-person investigations that incorporate the perspectives of patients and health professionals, improving understanding of the relationship between care provision, access, and effectiveness of care actions. The results point to the need for structural and organizational strengthening of PHC throughout the Brazilian national territory, with increased and reoriented public funding, incorporating best practices aimed at improving PHC team performance in line with the health actions examined in this study, the continuing education activities of teams, and the improvement of work processes ^18^.

The geographic region, population size, degree of social vulnerability, and FHS coverage of municipalities interacted with the structural and organizational characteristics of the BHUs, significantly influencing the completeness of actions. Municipalities with greater FHS coverage and higher social vulnerability showed higher completeness of care provision, highlighting the protective and equitable role of the FHS care model. Strengthening the FHS and consolidating multidisciplinary work have the potential to reduce long-standing inequalities, expand access, ensure comprehensive care, and increase PHC’s capacity to resolve health needs, thereby consolidating PHC as a fundamental pillar of the effectiveness and equity of the SUS.

## Data Availability

The sources of information used in the study are indicated in the body of the article.
