# Supplementary material for: Completeness of care provision and inequalities in care for people with hypertension, diabetes, and obesity: an analysis of the Brazilian National Basic Health Units Census
Source: Cad Saude Publica. 2026 Nov 2;42(Suppl 2):e00239325. doi: 10.1590/0102-311XEN239325 (PMC13730243; doi:10.1590/0102-311XEN239325)
Supplement: Translation

# Translation: Completude da oferta e desigualdades no cuidado a pessoas com hipertensão, diabetes e obesidade: uma análise do *Censo Nacional das Unidades Básicas de Saúde*

**DOI:** 10.1590/0102-311XPT239325

**Authors:** Luiz Augusto Facchini, Elaine Tomasi, Elaine Thumé, Fúlvio Borges Nedel, Bruna Venturin, Maria del Pilar Flores-Quispe, Vitória de Oliveira Ximendes, Michele Rohde Krolow, Anaclaudia Gastal Fassa

**Affiliations:** 1 Faculdade de Medicina, Universidade Federal de Pelotas, Pelotas, Brasil.; 2 Programa de Pós-graduação em Epidemiologia, Universidade Federal de Pelotas, Pelotas, Brasil.; 3 Faculdade de Enfermagem, Universidade Federal de Pelotas, Pelotas, Brasil.; 4 Departamento de Saúde Pública, Universidade Federal de Santa Catarina, Florianópolis, Brasil.; 5 Centro de Integração de Dados e Conhecimentos para Saúde, Fundação Oswaldo Cruz, Fiocruz, Salvador, Brasil.; 6 Secretaria de Vigilância em Saúde e Ambiente, Ministério da Saúde, Brasília, Brasil.; 7 Programa de Pós-graduação em Enfermagem, Universidade Federal de Pelotas, Pelotas, Brasil.

**Keywords:** Hipertensão, Diabetes Mellitus, Obesidade, Integralidade em Saúde, Saúde da Família

## Abstract

O estudo objetivou estabelecer padrões e desigualdades na completude da oferta de ações a pessoas com hipertensão arterial sistêmica, diabetes mellitus tipo 2 e obesidade na atenção primária à saúde (APS) brasileira, com base no *Censo Nacional das Unidades Básicas de Saúde* (Censo das UBS), realizado em 2024. Trata-se de um inquérito online, que abrangeu 44.938 UBS registradas no Cadastro Nacional de Estabelecimentos de Saúde e validadas pelos gestores municipais. Foram analisadas as variáveis relacionadas à região geopolítica, ao perfil dos municípios e aos serviços de saúde. A oferta de cuidados foi considerada completa quando a resposta foi positiva às 14 ações relacionadas à hipertensão, 15 à diabetes e 10 à obesidade. Observou-se uma baixa proporção de UBS com completude da oferta do cuidado a pessoas com hipertensão (15,3%), diabetes (15,6%) e obesidade (13,4%), em todas as regiões e perfis de município. A completude da oferta foi maior nas regiões Sudeste e Nordeste, e menor na Sul. Os grandes municípios e aqueles com vulnerabilidade social muito alta e alta, e com cobertura municipal de saúde da família de 100% também apresentaram maior completude. Após o ajuste para as características contextuais e das UBS, o modelo saúde da família aumentou em 70% a completude da oferta. Embora o acesso a consultas esteja consolidado, persistem fragilidades na integralidade e na coordenação do cuidado às doenças crônicas, exigindo políticas estruturantes que fortaleçam a capacidade resolutiva da APS.

## Introdução

A integralidade constitui um princípio estruturante do Sistema Único de Saúde (SUS), ao lado da universalidade e da equidade, abrangendo ações de cuidado individual e coletivo desde o domicílio, e a atenção primária à saúde (APS) até os serviços especializados e hospitalares. Inclui, ainda, intervenções de promoção da saúde, prevenção de agravos e vigilância, bem como a articulação intersetorial com áreas como educação, trabalho e assistência social ^1^.

A operacionalização e a avaliação da integralidade permanecem um desafio global, especialmente instigante frente à crescente ocorrência de condições crônicas de saúde ^2^. As doenças crônicas não transmissíveis (DCNT), como hipertensão, diabetes e obesidade, tornaram-se o principal desafio de saúde pública global no século XXI, sendo responsáveis por cerca de 75% de todas as mortes no mundo ^3^. As DCNT contribuem significativamente para mortes prematuras em todo o mundo, diminuindo também a qualidade de vida em geral ^4^.

No Brasil, as DCNT lideram as causas de óbitos, apesar do declínio observado entre 2000 e 2019 ^5^. Estima-se que, em 2030, o país apresente uma taxa de mortalidade prematura por DCNT de 274,0/100 mil habitantes, permanecendo acima da meta dos Objetivos de Desenvolvimento Sustentável (ODS) que prevê a redução em um terço desse indicador até 2030 (225,0/100 mil habitantes) ^6^.

A prevalência de hipertensão arterial sistêmica atinge 24% da população geral e chega a 61% entre idosos, e o diabetes mellitus apresenta prevalência de 9% na população geral e 23% nos maiores de 60 anos ^7^ ^,^ ^8^. A obesidade é um dos fatores de risco relevante para doenças cardiovasculares, diretamente associada à ocorrência de hipertensão e diabetes ^9^ ^,^ ^10^. Dados do Sistema de Vigilância de Fatores de Risco e Proteção para Doenças Crônicas por Inquérito Telefônico (Vigitel) informam que o excesso de peso vem crescendo, chegando a 58,8% em 2021, a mesma tendência da obesidade, atingindo 22,1% dos brasileiros no mesmo ano ^11^. Achados da *Pesquisa Nacional de Saúde* (PNS) de 2013 e 2019 corroboram o aumento das prevalências de obesidade e excesso de peso na população, passando de 20,8% para 25,9% e de 57% para 60,3%, respectivamente ^12^. A obesidade está associada ao aumento de atendimento médico em pronto-socorro e em unidades básicas de saúde (UBS), mesmo após ajuste para as variáveis sociodemográficas e sedentarismo ^13^.

As DCNT exercem um impacto substancial nos sistemas de saúde, exigindo cuidados complexos ao longo do tempo ^3^ ^,^ ^4^. Diferentes modelos têm sido adotados ao redor do mundo para responder às necessidades das pessoas afetadas, como por exemplo, o *Chronic Care Model* ^14^, amplamente utilizado para orientar a organização do cuidado, integrando componentes como suporte à decisão clínica, sistemas de informação, articulação comunitária e autocuidado apoiado. Suas características refletem os sistemas de saúde em que estão inseridas e as restrições de recursos para sua plena implementação ^15^ ^,^ ^16^.

No SUS, a Estratégia Saúde da Família (ESF) tem dedicado crescente esforço no enfrentamento da hipertensão arterial sistêmica, do diabetes mellitus tipo 2 e da obesidade. Essas condições compartilham fatores de risco modificáveis, como o sedentarismo, a alimentação inadequada e o excesso de peso, mas frequentemente coexistem, ampliando o dano à saúde, à complexidade clínica e aos custos do cuidado. A implementação de cuidados integrais às três condições crônicas requer ações abrangentes e completas de promoção da saúde, prevenção de agravos, tratamento, reabilitação e cuidados paliativos ^17^ ^,^ ^18^.

Entre 2012 e 2018, a avaliação externa do Programa de Melhoria do Acesso e da Qualidade da Atenção Básica (PMAQ-AB) evidenciou avanços no cuidado às DCNT. Houve aumento consistente na solicitação de exames laboratoriais, como creatinina, eletrocardiograma e perfil lipídico, na oferta de insumos específicos, como glicosímetros e tiras reagentes para glicemia capilar, e na programação de consultas periódicas para o acompanhamento de pessoas com DCNT ^19^. Ainda assim, apenas 35% das equipes de APS apresentavam organização considerada adequada para o cuidado a usuários com hipertensão e diabetes ^20^.

Do ponto de vista da experiência do usuário, também ocorreram fragilidades no cuidado integral, fundamental para responder a um conjunto complexo de necessidades e articular diferentes saberes e práticas no processo de trabalho. Apenas 31% dos pacientes com diabetes relataram a realização do exame dos pés - medida essencial para a prevenção de complicações - e menos de 20% receberam todos os componentes considerados parte de um cuidado integral ^19^ ^,^ ^20^. Apesar de avanços institucionais e normativos voltados à sua prevenção e controle, a carga das DCNT permanece elevada, gerando impactos significativos sobre a qualidade de vida da população e sobre a sustentabilidade do SUS, do qual depende a maioria dos brasileiros.

Na expectativa de contribuir para o enfrentamento dos desafios na operacionalização e avaliação da integralidade, este artigo objetivou analisar a completude da oferta de cuidados às pessoas com hipertensão, diabetes e obesidade na APS e identificar desigualdades associadas ao contexto dos municípios e às características das UBS, com base nos resultados do *Censo Nacional das Unidades Básicas de Saúde* (Censo das UBS) de 2024.

## Métodos

O Censo das UBS é um inquérito transversal, realizado de forma online entre junho e setembro de 2024, e foi liderado pelo Ministério da Saúde com o objetivo de produzir um diagnóstico detalhado da estrutura e da oferta de ações na APS e subsidiar o planejamento e a tomada de decisão na APS ^21^.

O universo do estudo abrangeu a totalidade das UBS registradas no Cadastro Nacional de Estabelecimentos de Saúde (CNES) em maio de 2024 e validadas pelos gestores municipais. A coleta de dados foi realizada por meio de questionário eletrônico estruturado, autodeclarado pelas equipes das UBS, com controle de qualidade e validação técnica. Detalhes sobre o desenvolvimento metodológico, a concepção dos instrumentos e a execução estão disponíveis no e-book *Panorama da APS no SUS: Censo Nacional das UBS, 2024* ^21^ e no artigo de Bousquat et al. ^22^.

### Características contextuais e das UBS

Neste estudo, as características contextuais e das UBS foram consideradas variáveis independentes. Esta seleção foi subsidiada por evidências sobre a relevância e a hierarquia das relações entre as variáveis contextuais em estudos de avaliação da APS, na conformação de padrões de qualidade na oferta de cuidados integrais às condições crônicas de saúde e na explicação de suas diferenças entre as UBS do país ^19^ ^,^ ^23^ ^,^ ^24^ ^,^ ^25^.

As características contextuais mais distais referem-se às regiões geopolíticas Norte, Nordeste, Sudeste, Sul e Centro-oeste e ao perfil dos municípios, classificados segundo porte populacional, Índice de Vulnerabilidade Social (IVS) ^26^ e cobertura da ESF. O porte populacional categorizou os municípios em até 20.000; de 20.001 a 50.000; de 50.001 a 100.000; de 100.001 a 900.000 e acima de 900.000 habitantes. O IVS estratifica os municípios nos níveis de vulnerabilidade muito alta, alta, média, baixa e muito baixa. A cobertura de ESF de 2023 expressa o percentual da população do município coberta por equipes deste modelo de atenção, agrupada em quatro categorias: abaixo de 50%, entre 50% e 74,9%, entre 75% e 99,9% e 100% ^27^.

As UBS foram caracterizadas quanto ao modelo de atenção, à estrutura física, ao perfil da equipe, à estrutura tecnológica e aos aspectos organizacionais. O modelo de atenção identificou a presença de pelo menos uma equipe de ESF na UBS (sim, não). A estrutura física avaliou a disponibilidade de oito itens essenciais para o cuidado de pessoas com hipertensão, diabetes ou obesidade: (i) estetoscópio para adultos, (ii) esfigmomanômetro para adultos, (iii) esfigmomanômetro para pessoas obesas, (iv) glicosímetro, (v) tiras reagentes para glicemia capilar, (vi) fita métrica, (vii) oxímetro digital e (viii) balança até 200kg - mecânica ou digital, todos de forma dicotômica (sim, não).

O perfil da equipe multiprofissional da UBS registrou a presença de quatro ou mais agentes comunitários de saúde (ACS) (sim, não), de profissionais da equipe multiprofissional (eMulti) (sim, não) e de profissionais de medicina e enfermagem com especialização em Medicina de Família e Comunidade (MFC) ou Saúde da Família (sim, não). A estrutura tecnológica foi examinada pelo o acesso adequado da UBS à internet (sim, não) e pela utilização de prontuário eletrônico (sim, não).

Os aspectos organizacionais da oferta de serviços pela UBS foram caracterizados pela participação da equipe em processos de educação permanente (sempre ou quase sempre, às vezes ou nunca), pela utilização de critérios de vulnerabilidade e risco para cadastramento populacional (sim, não) e pela existência de conselho local de saúde em atividade (sim, não).

### Oferta de cuidado a pessoas com hipertensão, diabetes e obesidade na APS

Para operacionalizar o conceito de atenção integral ^1^ ^,^ ^25^, os três desfechos do estudo foram caracterizados pela completude da oferta do cuidado nas UBS. Considerou-se um conjunto de ações de saúde comuns e específicas a cada agravo da tríade de condições crônicas, incluindo consultas, visita domiciliar, grupos educativos, lista de usuários com a condição, práticas corporais e atividade física, orientações, registro de parâmetros, estratificação de risco e solicitação de exames. A oferta de cuidados a cada um dos agravos foi considerada completa quando a resposta foi positiva para todas as ações avaliadas. No caso da hipertensão, foram 14 ações; no caso do diabetes, 15 ações; e para obesidade, 10 ações (Quadro 1), disponibilizadas no e-book ^21^ para o Brasil e grandes regiões.

**Quadro 1:** Ações selecionadas para análise da completude do cuidado a pessoas com hipertensão, diabetes e obesidade. *Censo Nacional das Unidades Básicas de Saúde*, Brasil, 2024.

	HIPERTENSÃO	DIABETES	OBESIDADE
Consulta médica para acompanhamento			
Consulta de enfermagem como parte do cuidado contínuo			
Visita domiciliar do ACS para busca ativa de usuários de maior risco ou que faltaram às consultas			
Atividades coletivas em grupos educativos como parte do cuidado contínuo			
Lista atualizada de pessoas com diagnóstico para acompanhamento			
Lista atualizada de usuários encaminhados a outros pontos da rede de atenção à saúde para acompanhamento			
Práticas corporais e atividades físicas promovidas nas UBS ou no território			
Oferta de orientações sobre alimentação adequada e saudável, com base no *Guia Alimentar para a População Brasileira*			
Verificação e registro da pressão arterial como rotina de acompanhamento e controle da hipertensão			
Avaliação do estado nutricional (peso e altura) com registro no prontuário clínico			
Acompanhamento do consumo alimentar com os marcadores de consumo alimentar no prontuário eletrônico			
Estratificação de risco cardiovascular com registro no prontuário clínico			
Solicitação de creatinina com registro no prontuário clínico			
Solicitação de perfil lipídico com registro no prontuário clínico			
Solicitação de ECG com registro no prontuário clínico			
Solicitação de glicemia de jejum com registro no prontuário clínico			
Solicitação de hemoglobina glicada com registro no prontuário clínico			
Exame dos pés como parte do cuidado contínuo			
Orientação para cuidado com os pés como parte do cuidado contínuo			
Orientação para uso de insulina como parte do cuidado contínuo			

ACS: agentes comunitários de saúde; UBS: unidades básicas de saúde.

Fonte: elaboração própria, com base nas informações publicadas no e-book *Panorama da APS no SUS: Censo Nacional das UBS, 2024* ^21^.

A seleção das ações de saúde levou em conta a relevância clínica e programática no enfrentamento das DCNT na APS, com base em documentos oficiais ^28^ ^,^ ^29^ ^,^ ^30^ ^,^ ^31^ ^,^ ^32^, protocolos e evidências de estudos, que aprofundaram a compreensão sobre a caracterização do cuidado integral e resolutivo à população e aos usuários das UBS do país ^19^ ^,^ ^23^ ^,^ ^24^ ^,^ ^25^ ^,^ ^31^ ^,^ ^32^. Utilizou-se para a análise de completude a totalidade das variáveis disponíveis no instrumento para cada agravo. No processo de elaboração do instrumento, o escopo das ações foi definido pelas áreas técnicas do Ministério da Saúde, embasado em protocolos e documentos oficiais.

### Modelo teórico, categorias e procedimentos de análise

Foram realizadas análises descritivas para estimar as frequências absolutas e relativas de cada uma das ações que compõe a caracterização da completude da oferta de cuidados, bem como da própria completude, em cada uma das condições - hipertensão, diabetes e obesidade - nas UBS do país.

A análise multivariada utilizou uma abordagem hierárquica ^3^ ^,^ ^24^ multinível ^33^ para ajustar o efeito do contexto e das UBS sobre a completude da oferta de cuidados às três condições crônicas. A opção reconhece a dependência direta das UBS (nível 1) dos contextos municipais (nível 2) nos quais estão inseridas, expressa na correlação intraclasse entre serviços de APS de um mesmo município. A modelagem é indispensável diante da complexa estrutura e assimetria dos dados, em que municípios de pequeno porte frequentemente têm uma única UBS, enquanto grandes centros urbanos dispõem de dezenas ou centenas de UBS. Ao controlar a correlação intraclasse (ICC) decorrente desse aninhamento contextual, a abordagem corrige a dependência dos dados e assegura estimativas estatísticas mais precisas e válidas sobre a oferta de cuidado. No nível 1, os resultados de cada variável foram ajustados para todas as variáveis das UBS (nível 1) e todas as variáveis do contexto (nível 2). No nível 2, os resultados de cada variável foram ajustados para todas as variáveis contextuais.

O modelo multinível utilizou a regressão de Poisson com variância robusta, estimando razões de prevalência (RP) brutas e ajustadas e os respectivos intervalos de 95% de confiança (IC95%). Estimou-se o valor de p com o teste de Wald de heterogeneidade para as variáveis categóricas e de tendência linear para as variáveis contínuas, considerando-se significativo o valor de p < 0,05. Todas as análises foram realizadas no Stata, versão 17.0 (https://www.stata.com).

### Aspectos éticos

O protocolo de pesquisa foi avaliado e aprovado pelo Comitê de Ética em Pesquisa da Faculdade de Saúde Pública, Universidade de São Paulo (parecer nº 6.779.222, emitido em 25 de abril de 2024, CAAE: 78767024.2.0000.5421).

## Resultados

### Distribuição das UBS de acordo com fatores contextuais

O Censo das UBS de 2024 incluiu 44.938 UBS em todo o território nacional. A maior concentração de UBS ocorreu no Nordeste (39,5%), seguido pelo Sudeste (29,7%) e o Sul (14,7%), já o Norte (9,1%) e o Centro-oeste (7%) apresentaram os menores quantitativos ^21^ ^,^ ^22^.

Quanto ao porte populacional, 34,6% das UBS estavam situadas em municípios de até 20.000 habitantes, 25,4% em municípios entre 20.001 e 50.000 habitantes, 13,4% em municípios de 50.001 a 100.000 habitantes, 21,5% em centros urbanos de 100.001 a 900.000 habitantes e 5,2% em municípios acima de 900.000 habitantes. A distribuição das UBS segundo o IVS revelou que mais de um terço encontrava-se em municípios com alta (21,4%) ou muito alta vulnerabilidade (15,1%), 23,5% com média vulnerabilidade, 29,7% com vulnerabilidade baixa e 10,2% com muito baixa vulnerabilidade social. A distribuição das UBS de acordo com a cobertura municipal de ESF indica que a maior parte (59,3%) está em municípios com 100% de cobertura, 17,9% em municípios entre 75% e 99,9%, e 14,4% em municípios com cobertura entre 50% e 74,9% ^21^.

### Distribuição das UBS segundo características dos serviços

Em 88,5% das UBS havia pelo menos uma equipe da ESF e 32,4% dispunham de todos os oito itens de equipamentos e insumos avaliados. Em relação ao perfil da equipe das UBS, 74,7% relataram a presença de quatro ou mais ACS, 42,2% recebiam apoio de equipes eMulti e 17,3% contavam com profissionais de medicina e enfermagem com especialização em MFC ou Saúde da Família. O acesso adequado à internet foi referido por 65,2% e a utilização de prontuário eletrônico foi informada por 87,3% das UBS. No que se refere às características organizacionais das UBS, 64,8% confirmaram a participação da equipe em processos de educação permanente sempre ou quase sempre, 59,9% utilizavam critérios de vulnerabilidade e risco para cadastramento populacional e 36,3% tinham conselho local de saúde em atividade ^21^.

### Oferta de ações de saúde para pessoas com hipertensão, diabetes e obesidade

As ações com prevalências superiores a 80% das UBS foram consultas médicas e de enfermagem para todas as condições; visita domiciliar do ACS para hipertensão e diabetes; verificação e registro da pressão arterial e a solicitação de exame de creatinina, perfil lipídico e ECG para hipertensão; solicitação de glicemia de jejum e hemoglobina glicada para diabetes; e avaliação de peso e altura para obesidade (Tabela 1).

**Tabela 1:** Proporção de unidades básicas de saúde (UBS) do Sistema Único de Saúde (SUS) que ofertam as ações de saúde no cuidado de pessoas com hipertensão arterial sistêmica, diabetes mellitus tipo 2 e obesidade. *Censo Nacional das Unidades Básicas de Saúde*, 2024 (N = 44.938).

Ações de saúde *	Hipertensão		Diabetes		Obesidade	
	n	%	n	%	n	%
Consulta médica	43.828	97,5	43.881	97,7	41.733	92,9
Consulta de enfermagem	42.136	93,8	42.252	94,0	37.845	84,2
Visita domiciliar do ACS	38.323	85,3	37.661	83,8	31.124	69,3
Atividades coletivas em grupos educativos	30.114	67,0	28.671	63,8	19.728	43,9
Lista atualizada de pessoas com o agravo	34.227	76,2	33.985	75,6	16.670	37,1
Lista atualizada de pessoas com o agravo encaminhadas a outros pontos da rede de atenção à saúde	19.589	43,6	19.820	44,1	12.365	27,5
Práticas corporais e atividades físicas	19.300	43,0	19.258	42,9	17.536	39,0
Orientação sobre alimentação adequada e saudável	35.088	78,1	33.110	73,7	25.948	57,8
Verificação e registro da pressão arterial	43.699	97,3	-	-	-	-
Avaliação do estado nutricional (peso e altura)	-	-	31.150	69,3	38.522	85,7
Acompanhamento do consumo alimentar	22.328	49,7	-	-	24.894	55,4
Estratificação de risco cardiovascular	27.539	61,3	29.011	64,6	-	-
Solicitação de exame de creatinina	39.221	87,3	-	-	-	-
Solicitação de exame de perfil lipídico	37.069	82,5	-	-	-	-
Solicitação de exame ECG	38.807	86,4	-	-	-	-
Solicitação de glicemia de jejum	-	-	42.577	94,8	-	-
Solicitação de hemoglobina glicada	-	-	42.528	94,6	-	-
Exame dos pés	-	-	26.354	58,7	-	-
Orientação para autocuidado com os pés	-	-	35.019	77,9	-	-
Orientação para uso de insulina	-	-	38.743	86,2	-	-

ACS: agentes comunitários de saúde.

* Informações publicadas no e-book *Panorama da APS no SUS: Censo Nacional das UBS, 2024* ^21^.

Prevalências entre 51% e 79% das UBS foram observadas para orientação sobre alimentação adequada e saudável para todas as condições; atividades coletivas em grupos educativos e lista atualizada de pessoas com o agravo; estratificação do risco cardiovascular; avaliação de peso e altura para hipertensão e diabetes; exame dos pés e orientação para o autocuidado dos pés para pessoas com diabetes; e visita domiciliar do ACS e acompanhamento do consumo alimentar para obesidade (Tabela 1).

Já prevalências entre 27% e 50% das UBS foram registradas para manter a lista atualizada de pessoas com o agravo encaminhadas a outros pontos da rede de atenção, práticas corporais e atividades físicas para todas as condições; acompanhamento do consumo alimentar para hipertensão; e atividades coletivas em grupos educativos e lista atualizada de pessoas com o agravo para obesidade (Tabela 1).

### Completude na oferta do cuidado às pessoas com hipertensão, diabetes e obesidade

As Tabelas 2, 3 e 4 (hipertensão, diabetes e obesidade, respectivamente) apresentam a distribuição da completude na oferta de ações e as associações com as características contextuais e das UBS, por meio de análises multivariadas, brutas e ajustadas. No Brasil, 15,3% (n = 6.863) das UBS ofereciam cuidado completo às pessoas com hipertensão, 15,6% (n = 7.008) com diabetes e 13,4% (n = 6.018) com obesidade.

**Tabela 2:** Completude da oferta de ações de cuidado a pessoas com hipertensão arterial sistêmica, segundo a região, o perfil dos municípios e as características das unidades básicas de saúde (UBS). *Censo Nacional das Unidades Básicas de Saúde*, 2024 (N = 44.938).

	Frequência de completude		Bruta		Ajustada *	
	n	%	RP	IC95%	RP	IC95%
Características do contexto (nível 2)						
Região				p < 0,001		p = 0,001
Centro-oeste	412	13,2	1,36	1,12-1,65	1,23	1,04-1,46
Nordeste	2.900	16,4	1,74	1,52-1,99	1,19	1,01-1,40
Norte	544	13,3	1,36	1,13-1,63	1,06	0,87-1,28
Sudeste	2.376	17,8	1,67	1,45-1,92	1,52	1,34-1,72
Sul	631	9,6	Referência		Referência	
Porte populacional (habitantes)				p = 0,004		p = 0,001
Acima de 900.000	658	28,0	1,66	1,12-2,44	1,91	1,36-2,68
De 100.001 a 900.000	1.310	13,6	0,91	0,79-1,05	1,18	1,01-1,39
De 50.001 a 100.000	877	14,6	0,92	0,80-1,07	1,09	0,95-1,26
De 20.001 a 50.000	1.596	14,0	0,87	0,79-0,97	0,95	0,86-1,04
Até 20.000	2.422	15,6	Referência		Referência	
Índice de Vulnerabilidade Social				p < 0,001		p = 0,001
Muito alta	1.081	16,0	1,47	1,23-1,75	1,37	1,12-1,68
Alta	1.651	17,1	1,61	1,36-1,90	1,37	1,13-1,64
Média	1.570	14,9	1,35	1,14-1,59	1,14	0,97-1,34
Baixa	2.038	15,3	1,18	1,00-1,39	1,01	0,87-1,17
Muito baixa	523	11,4	Referência		Referência	
Cobertura de Saúde da Família (%)				p < 0,001		p < 0,001
Abaixo de 50	463	12,2	Referência		Referência	
Entre 50 e 74,9	931	14,4	1,73	1,31-2,28	1,55	1,21-2,00
Entre 75 e 99,9	1.109	13,8	1,99	1,54-2,58	1,99	1,55-2,56
100	4.360	16,4	2,65	2,08-3,36	2,40	1,85-3,13
Características das UBS (nível 1)						
Modelo de atenção				p < 0,001		p < 0,001
ESF	6.564	16,5	2,90	2,31-3,62	1,69	1,36-2,11
Outro	287	5,6	Referência		Referência	
Equipamentos e insumos (8 itens **)				p < 0,001		p < 0,001
Sim	2.823	19,4	1,34	1,28-1,41	1,25	1,19-1,31
Não	4.039	13,3	Referência		Referência	
UBS com 4 ou mais ACS				p < 0,001		p < 0,001
Sim	5.876	17,5	1,87	1,65-2,10	1,26	1,16-1,38
Não	985	8,7	Referência		Referência	
UBS com eMulti				p < 0,001		p < 0,001
Sim	3.940	20,8	1,58	1,48-1,68	1,31	1,24-1,39
Não	2.923	11,3	Referência		Referência	
Médico e enfermeiro com especialização em MFC/Saúde da Família				p < 0,001		p < 0,001
Sim	1.575	20,3	1,33	1,26-1,41	1,18	1,11-1,25
Não	5.286	14,2	Referência		Referência	
Acesso adequado à internet na UBS				p < 0,001		p = 0,010
Sim	4.925	16,8	1,21	1,14-1,28	1,07	1,02-1,13
Não	1.937	12,4	Referência		Referência	
UBS utiliza prontuário eletrônico				p < 0,001		p < 0,001
Sim	6.361	16,2	1,81	1,63-2,02	1,38	1,23-1,53
Não	501	8,8	Referência		Referência	
Profissionais participam de educação permanente				p < 0,001		p < 0,001
Sempre ou quase sempre	5.746	19,7	2,34	2,18-2,52	1,94	1,81-2,09
Às vezes ou nunca	1.116	7,1	Referência		Referência	
UBS utiliza critério de vulnerabilidade e risco para cadastramento populacional				p < 0,001		p < 0,001
Sim	5.500	20,4	2,27	2,10-2,46	1,87	1,72-2,04
Não	1.361	7,6	Referência		Referência	
Conselho local de saúde ativo				p < 0,001		p < 0,001
Sim	3.553	21,8	1,65	1,56-1,74	1,41	1,33-1,49
Não	3.308	11,6	Referência		Referência	
Total	6.863	15,3				

ACS: agentes comunitários de saúde; ESF: Estratégia Saúde da Família; eMulti: equipes multiprofissionais; IC95%: intervalo de 95% de confiança; MFC: Medicina de Família e Comunidade; RP: razão de prevalência.

* No nível 1, os resultados de cada variável foram ajustados para todas as variáveis das características das UBS e todas as variáveis do nível 2, características do contexto. No nível 2, os resultados de cada variável foram ajustados para todas as variáveis das características do contexto;

** Oito itens essenciais para o cuidado de pessoas com hipertensão, diabetes ou obesidade: (i) estetoscópio para adultos, (ii) esfigmomanômetro para adultos, (iii) esfigmomanômetro para pessoas obesas, (iv) glicosímetro, (v) tiras reagentes para glicemia capilar, (vi) fita métrica, (vii) oxímetro digital e (viii) balança até 200kg - mecânica ou digital.

**Tabela 3:** Completude da oferta de ações de cuidado a pessoas com diabetes mellitus, segundo a região, o perfil dos municípios e as características das unidades básicas de saúde (UBS). *Censo Nacional das Unidades Básicas de Saúde*, 2024 (N = 44.938).

	Frequência de completude		Bruta		Ajustada *	
	n	%	RP	IC95%	RP	IC95%
Características do contexto (nível 2)						
Região				p < 0,001		p = 0,001
Centro-oeste	383	12,3	1,11	0,91-1,34	1,02	0,86-1,22
Nordeste	2.953	16,7	1,55	1,36-1,77	1,11	0,95-1,30
Norte	521	12,7	1,08	0,90-1,31	0,87	0,72-1,06
Sudeste	2.442	18,3	1,45	1,26-1,66	1,33	1,18-1,51
Sul	709	10,7	Referência		Referência	
Porte populacional (habitantes)				p = 0,008		p = 0,001
Acima de 900.000	725	30,9	1,86	1,25-2,77	2,15	1,52-3,05
De 100.001 a 900.000	1.425	14,8	1,03	0,90-1,18	1,34	1,14-1,57
De 50.001 a 100.000	845	14,1	0,91	0,78-1,05	1,08	0,94-1,24
De 20.001 a 50.000	1.662	14,6	0,93	0,84-1,04	1,02	0,92-1,12
Até 20.000	2.351	15,1	Referência		Referência	
Índice de Vulnerabilidade Social				p < 0,001		p = 0,006
Muito alta	1.071	15,8	1,32	1,11-1,57	1,25	1,02-1,52
Alta	1.611	16,7	1,40	1,19-1,65	1,22	1,01-1,46
Média	1.627	15,4	1,23	1,04-1,45	1,08	0,92-1,27
Baixa	2.116	15,8	1,06	0,90-1,25	0,94	0,82-1,09
Muito baixa	583	12,7	Referência		Referência	
Cobertura de Saúde da Família (%)				p < 0,001		p = 0,001
Abaixo de 50	533	14,1	Referência		Referência	
Entre 50 e 74,9	994	15,4	1,70	1,29-2,24	1,52	1,18-1,95
Entre 75 e 99,9	1.143	14,2	1,83	1,41-2,36	1,86	1,46-2,37
100	4.338	16,3	2,41	1,90-3,06	2,32	1,80-2,98
Características das UBS (nível 1)						
Modelo de atenção				p < 0,001		p < 0,001
ESF	6.703	16,9	2,90	2,32-3,64	1,71	1,38-2,10
Outro	295	5,7	Referência		Referência	
Equipamentos e insumos (8 itens **)				p < 0,001		p < 0,001
Sim	2.938	20,2	1,36	1,29-1,43	1,26	1,20-1,33
Não	4.069	13,4	Referência		Referência	
UBS com 4 ou mais ACS				p < 0,001		p < 0,001
Sim	6.033	18,0	1,94	1,71-2,20	1,35	1,23-1,47
Não	974	8,6	Referência		Referência	
UBS com eMulti				p < 0,001		p < 0,001
Sim	4.046	21,3	1,60	1,51-1,70	1,35	1,27-1,42
Não	2.962	11,4	Referência		Referência	
Médico e enfermeiro com especialização em MFC/Saúde da Família				p < 0,001		p < 0,001
Sim	1.620	20,8	1,30	1,22-1,37	1,14	1,08-1,21
Não	5.385	14,5	Referência		Referência	
Acesso adequado à internet na UBS				p < 0,001		p < 0,001
Sim	5.042	17,2	1,22	1,16-1,29	1,09	1,04-1,15
Não	1.964	12,5	Referência		Referência	
UBS utiliza prontuário eletrônico				p < 0,001		p < 0,001
Sim	6.429	16,4	1,59	1,44-1,76	1,17	1,06-1,30
Não	577	10,1	Referência		Referência	
Profissionais participam de educação permanente				p < 0,001		p < 0,001
Sempre ou quase sempre	5.864	20,1	2,26	2,11-2,43	1,89	1,77-2,03
Às vezes ou nunca	1.143	7,2	Referência		Referência	
UBS utiliza critério de vulnerabilidade e risco para cadastramento populacional				p < 0,001		p < 0,001
Sim	5.552	20,6	2,16	2,00-2,33	1,79	1,66-1,94
Não	1.454	8,1	Referência		Referência	
Conselho local de saúde ativo				p < 0,001		p < 0,001
Sim	3.600	22,1	1,58	1,49-1,67	1,36	1,29-1,44
Não	3.406	11,9	Referência		Referência	
Total	7.008	15,6				

ACS: agentes comunitários de saúde; ESF: Estratégia Saúde da Família; eMulti: equipes multiprofissionais; IC95%: intervalo de 95% de confiança; MFC: Medicina de Família e Comunidade; RP: razão de prevalência.

* No nível 1, os resultados de cada variável foram ajustados para todas as variáveis das características das UBS e todas as variáveis do nível 2, características do contexto. No nível 2, os resultados de cada variável foram ajustados para todas as variáveis das características do contexto;

** Oito itens essenciais para o cuidado de pessoas com hipertensão, diabetes ou obesidade: (i) estetoscópio para adultos, (ii) esfigmomanômetro para adultos, (iii) esfigmomanômetro para pessoas obesas, (iv) glicosímetro, (v) tiras reagentes para glicemia capilar, (vi) fita métrica, (vii) oxímetro digital e (viii) balança até 200kg - mecânica ou digital.

**Tabela 4:** Completude da oferta de ações de cuidado a pessoas com obesidade, segundo a região, o perfil dos municípios e as características das unidades básicas de saúde (UBS). *Censo Nacional das Unidades Básicas de Saúde*, 2024 (N = 44.938).

	Frequência de completude		Bruta		Ajustada *	
	n	%	RP	IC95%	RP	IC95%
Características do contexto (nível 2)						
Região				p < 0,001		p = 0,001
Centro-oeste	340	10,9	1,39	1,13-1,72	1,27	1,05-1,54
Nordeste	2.705	15,3	2,02	1,75-2,34	1,47	1,23-1,75
Norte	482	11,8	1,48	1,21-1,81	1,22	0,99-1,51
Sudeste	1.993	14,9	1,70	1,46-1,99	1,58	1,37-1,81
Sul	498	7,5	Referência		Referência	
Porte populacional (habitantes)				p = 0,014		p = 0,951
Acima de 900.000	541	23,0	1,18	0,65-2,11	1,17	0,68-2,02
De 100.001 a 900.000	1.044	10,8	0,80	0,69-0,93	0,99	0,83-1,17
De 50.001 a 100.000	725	12,1	0,82	0,70-0,97	0,96	0,83-1,12
De 20.001 a 50.000	2.191	14,1	0,92	0,82-1,03	1,00	0,91-1,11
Até 20.000	1.517	13,3	Referência		Referência	
Índice de Vulnerabilidade Social				p < 0,001		p = 0,037
Muito alta	980	14,5	1,61	1,33-1,95	1,20	0,96-1,50
Alta	1.542	16,0	1,79	1,49-2,14	1,25	1,02-1,54
Média	1.397	13,2	1,44	1,20-1,74	1,13	0,94-1,35
Baixa	1.685	12,6	1,18	0,98-1,42	0,98	0,83-1,15
Muito baixa	414	9,0	Referência		Referência	
Cobertura de Saúde da Família (%)				p < 0,001		p < 0,001
Abaixo de 50	389	10,3	Referência		Referência	
Entre 50 e 74,9	792	12,2	1,73	1,22-2,44	1,71	1,29-2,27
Entre 75 e 99,9	887	11,1	1,87	1,36-2,56	1,85	1,40-2,44
100	3.950	14,8	2,77	2,07-3,71	2,30	1,72-3,06
Características das UBS (nível 1)						
Modelo de atenção				p < 0,001		p < 0,001
ESF	5.764	14,5	2,98	2,40-3,69	1,75	1,42-2,16
Outro	241	4,7	Referência		Referência	
Equipamentos e insumos (8 itens **)				p < 0,001		p < 0,001
Sim	2.462	16,7	1,37	1,29-1,45	1,28	1,21-1,36
Não	3.554	11,7	Referência		Referência	
UBS com 4 ou mais ACS				p < 0,001		p < 0,001
Sim	5.127	15,3	1,81	1,59-2,05	1,20	1,10-1,31
Não	889	7,8	Referência		Referência	
UBS com eMulti				p < 0,001		p < 0,001
Sim	3.533	18,6	1,69	1,59-1,80	1,40	1,32-1,48
Não	2.485	9,6	Referência		Referência	
Médico e enfermeiro com especialização em MFC/Saúde da Família				p < 0,001		p < 0,001
Sim	1.365	17,6	1,36	1,28-1,44	1,20	1,13-1,28
Não	4.651	12,5	Referência		Referência	
Acesso adequado à internet na UBS				p < 0,001		p = 0,004
Sim	4.356	14,9	1,23	1,16-1,31	1,09	1,03-1,16
Não	1.661	10,6	Referência		Referência	
UBS utiliza prontuário eletrônico				p < 0,001		p < 0,001
Sim	5.542	14,1	1,67	1,49-1,87	1,27	1,13-1,42
Não	475	8,3	Referência		Referência	
Profissionais participam de educação permanente				p < 0,001		p < 0,001
Sempre ou quase sempre	5.113	17,6	2,58	2,38-2,80	2,11	1,95-2,28
Às vezes ou nunca	904	5,7	Referência		Referência	
UBS utiliza critério de vulnerabilidade e risco para cadastramento populacional				p < 0,001		p < 0,001
Sim	4.872	18,1	2,41	2,20-2,64	1,96	1,79-2,15
Não	1.144	6,4	Referência		Referência	
Conselho local de saúde ativo				p < 0,001		p < 0,001
Sim	3.216	19,7	1,82	1,71-1,94	1,55	1,46-1,65
Não	2.800	9,8	Referência		Referência	
Total	6.018	13,4				

ACS: agentes comunitários de saúde; ESF: Estratégia Saúde da Família; eMulti: equipes multiprofissionais; IC95%: intervalo de 95% de confiança; MFC: Medicina de Família e Comunidade; RP: razão de prevalência.

* No nível 1, os resultados de cada variável foram ajustados para todas as variáveis das características das UBS e todas as variáveis do nível 2, características do contexto. No nível 2, os resultados de cada variável foram ajustados para todas as variáveis das características do contexto;

** Oito itens essenciais para o cuidado de pessoas com hipertensão, diabetes ou obesidade: (i) estetoscópio para adultos, (ii) esfigmomanômetro para adultos, (iii) esfigmomanômetro para pessoas obesas, (iv) glicosímetro, (v) tiras reagentes para glicemia capilar, (vi) fita métrica, (vii) oxímetro digital e (viii) balança até 200kg - mecânica ou digital.

### Completude de acordo com as características contextuais

Na análise multinível bruta, observou-se que as UBS da Região Nordeste apresentaram desempenho superior quanto à completude da oferta de ações. Em comparação à Região Sul, as UBS nordestinas exibiram maior probabilidade de completude do cuidado para hipertensão (RP = 1,74; IC95%: 1,52-1,99), diabetes (RP = 1,55; IC95%: 1,36-1,77) e obesidade (RP = 2,00; IC95%: 1,75-2,34). As UBS da Região Sudeste também se destacaram, com níveis de completude entre 45% e 70% superiores aos observados no Sul. Por sua vez, as regiões Centro-oeste e Norte apresentaram desempenho significativamente superior ao Sul no cuidado à hipertensão e à obesidade, já para diabetes, os resultados foram semelhantes entre essas regiões.

Após ajuste para as variáveis contextuais, houve uma atenuação das desigualdades regionais. Nessa etapa, as UBS do Sudeste passaram a apresentar maior padrão de completude, 50% superior ao das UBS do Sul. Embora a vantagem do Nordeste tenha diminuído, a região manteve diferenças significativas no cuidado à hipertensão e à obesidade, e padrão semelhante ao do Sul no cuidado à diabetes. As UBS do Centro-oeste apresentaram comportamento análogo às do Nordeste, e as do Norte passaram a exibir completudes similares às do Sul.

Na análise bruta, as UBS localizadas em municípios com mais de 900.000 habitantes apresentaram níveis de completude na oferta de cuidado à hipertensão e à diabetes significativamente maiores do que as dos demais portes populacionais. Ao ajustar para as variáveis contextuais, as UBS dos maiores municípios apresentaram probabilidade de completude para hipertensão e diabetes significativamente maior do que as de municípios de médio e pequeno porte. No caso da obesidade, a completude da oferta foi semelhante entre os diferentes portes populacionais, tanto na análise bruta quanto na ajustada.

As UBS de municípios com alta e muito alta vulnerabilidade social apresentaram, na análise bruta, padrões de completude da oferta 50% a 60% superiores para hipertensão, 30% a 40% para diabetes e 60% a 80% para obesidade, quando comparadas aos de municípios com menor vulnerabilidade. Após o ajuste para as variáveis contextuais, as UBS situadas em municípios com maior vulnerabilidade mantiveram completude significativamente maior - entre 20% e 40% - para as três condições analisadas. Já as UBS de municípios com IVS baixo e muito baixo apresentaram desempenho semelhante entre si, mas consistentemente inferior ao observado em contextos mais vulneráveis.

Verificou-se, ainda, uma relação positiva de tipo dose-resposta entre a cobertura da ESF e a completude da oferta de ações para a tríade de agravos. Essa associação persistiu após o ajuste para as variáveis contextuais: UBS localizadas em municípios com 100% de cobertura da ESF apresentaram o dobro de probabilidade de completude do que aquelas inseridas em municípios com cobertura inferior a 50%.

### Completude de acordo com características das UBS

Na análise bruta, as UBS com modelo de atenção da ESF apresentaram probabilidade quase três vezes maior de completude na oferta de ações voltadas ao cuidado da hipertensão, diabetes e obesidade, em comparação às UBS sem ESF. Após o ajuste para as variáveis de contexto e da UBS, o efeito do modelo de atenção reduziu, mas permaneceu significativo, com probabilidade aproximadamente 70% superior nas UBS com equipes de ESF.

A disponibilidade dos oito equipamentos e insumos essenciais avaliados também esteve associada positivamente à completude da oferta. Na análise bruta, essas UBS apresentaram proporções de completude cerca de 35% maiores para as três condições. A associação se manteve significativa após o ajuste: unidades com infraestrutura completa apresentaram probabilidade cerca de 25% superior àquelas que não dispunham da totalidade dos equipamentos.

No perfil da equipe, UBS com quatro ou mais ACS registraram, na análise bruta, proporções de completude entre 80% e 95% maiores que aquelas com menos ACS. Após o ajuste, a magnitude do efeito diminuiu, mas manteve-se estatisticamente significativa, com completude de 20% a 30% superior nas UBS com quatro ou mais ACS.

De forma semelhante, a presença de profissionais médicos e de enfermagem com especialização ou titulação em MFC ou Saúde da Família esteve associada a melhores resultados. Na análise bruta, a completude das ações foi de 30% a 40% maior nessas UBS e, após o ajuste, manteve-se de 15% a 20% superior em relação àquelas que não contavam com profissionais com essa formação.

As UBS que contavam com o apoio da eMulti apresentaram, na análise bruta, completude de ações entre 60% e 70% maior que aquelas sem esse suporte. Mesmo após o ajuste, manteve-se a associação significativa: unidades com apoio da eMulti registraram completude de 30% a 40% superior no cuidado à hipertensão, diabetes e obesidade.

O acesso à internet também foi relevante na análise bruta: UBS com conectividade adequada apresentaram proporções de completude cerca de 20% superiores às de unidades com acesso precário. Após o ajuste, a completude das ações foi maior em cerca de 10% para as UBS com acesso adequado à internet. A utilização do prontuário eletrônico foi associada a proporções 60% a 80% maiores na análise bruta. Após o ajuste, as UBS com essa ferramenta apresentaram completude de 20% a 40% superior àquelas sem prontuário eletrônico.

A qualificação das equipes também demonstrou efeito significativo. As UBS cujos profissionais participavam sempre ou quase sempre de atividades de educação permanente apresentaram, na análise bruta, padrões de completude de 2,0 a 2,6 vezes superiores aos das UBS com participação eventual ou inexistente. Após o ajuste, a associação permaneceu significativa, com completude de 90% a 110% maior nas UBS com equipes engajadas em processos regulares de educação permanente.

Outro destaque foi o uso de critérios de vulnerabilidade e risco para o cadastramento de usuários pelas UBS que, na análise bruta, apresentou probabilidade de completude entre 2,0 e 2,4 vezes superior à de UBS que não o faziam. A diferença permaneceu expressiva após o ajuste, com probabilidade de 80% a 96% maior de completude da oferta nas UBS que adotavam critérios de vulnerabilidade no cadastramento.

Por fim, a presença de um conselho local de saúde ativo esteve associada, na análise bruta, a proporções de completude entre 60% e 80% superiores às das unidades sem o dispositivo. Após o ajuste, a associação persistiu, sendo a probabilidade de completude de 35% a 55% maior nas UBS com conselho local de saúde em funcionamento.

A ICC estimada na análise multinível foi ≥ 0,80, indicando uma forte dependência das características contextuais dos municípios no efeito sobre a oferta de ações de saúde completas para os três agravos ^33^.

## Discussão

Os resultados do presente estudo sinalizam que cerca de 85% das UBS do SUS não alcançaram a completude da oferta de ações para o cuidado de pessoas com hipertensão, diabetes e obesidade. Também revelam uma variabilidade significativa do *proxy* de integralidade do cuidado, em função das características contextuais e dos próprios serviços de APS.

As ações clínicas básicas, como consultas e verificação de parâmetros biométricos, estão amplamente institucionalizadas na rotina das UBS para as três condições estudadas. A visita domiciliar de ACS para a busca ativa de usuários faltosos foi amplamente referida para hipertensão e diabetes, à semelhança do observado no primeiro ciclo do PMAQ ^19^, indicando a prioridade histórica desses agravos na APS, já o menor percentual relacionado à obesidade sugere sua menor integração às estratégias de cuidado.

A avaliação do estado nutricional foi mais modesta para diabetes, sugerindo fragilidade no monitoramento regular de parâmetros clínicos essenciais para pessoas com risco metabólico. Também houve baixa incorporação de práticas educativas, preventivas e interprofissionais. A obesidade, em especial, foi responsável por menores proporções de práticas educativas e orientação baseada no *Guia Alimentar para a População Brasileira* ^34^ ^,^ ^35^.

O número reduzido de UBS com listas de acompanhamento de usuários indica lacunas na vigilância longitudinal e na coordenação do cuidado, especialmente para obesidade. A oferta ainda limitada dessas ações sugere uma fragmentação do cuidado das três condições estudadas e o potencial subaproveitamento de estratégias preventivas para problemas que compartilham fatores de risco e curso simultâneo, configurando um dos perfis de multimorbidade mais frequentes ^36^ ^,^ ^37^ ^,^ ^38^.

As desigualdades na completude da oferta de cuidado à hipertensão, à diabetes e à obesidade refletem tanto características contextuais quanto dos serviços. As UBS das regiões Sudeste e Nordeste se destacaram com os maiores padrões de completude. O Sudeste reúne municípios mais populosos, com grande capacidade instalada de serviços de saúde, e o Nordeste agrega municípios com maior proporção de UBS com ESF. Logo, UBS equipadas e organizadas, com equipes da ESF completas e ação comunitária se associaram a proporções significativamente maiores de completude de ações de saúde no cuidado de pessoas com hipertensão, diabetes e obesidade. Essa situação é coerente com a maior completude das ações para hipertensão e diabetes ^20^ ^,^ ^38^ ^,^ ^39^, em grandes municípios, especialmente naqueles com mais de 900.000 habitantes e nos municípios com 100% de cobertura populacional da ESF.

O financiamento da APS tem beneficiado historicamente municípios com maior vulnerabilidade ^40^, havendo evidências de resultados mais equitativos em avaliações da APS, que corroboram os resultados das UBS de municípios mais vulneráveis ^41^. No Nordeste, o melhor desempenho estar associado à maior cobertura da ESF tem contribuído para a marcante superação de iniquidades, já perceptíveis no ciclo três do PMAQ ^20^ ^,^ ^38^ ^,^ ^39^ ^,^ ^41^ ^,^ ^42^. O crescimento da cobertura municipal da ESF mostrou forte associação com o aumento da completude da oferta. O Sul e o Norte, por razões distintas, mostraram as piores situações. O Norte é a região com a menor infraestrutura, as maiores dificuldades de acesso e provimento de profissionais. O Sul parece ter perdido o protagonismo na APS ao longo do tempo, com sua rede básica deixando de estar entre as melhores do país, situação já observada em 2018, no ciclo três do PMAQ ^20^ ^,^ ^38^ ^,^ ^39^ ^,^ ^41^ ^,^ ^43^. É importante registrar que a coleta de dados ocorreu em 2024, período em que o Rio Grande do Sul foi gravemente afetado por um evento climático extremo que prejudicou direta e indiretamente a estrutura das UBS e a capacidade de oferta de cuidados em saúde. Porém, a exclusão das UBS gaúchas da análise não foi suficiente para tirar a Região Sul do último lugar.

A maior completude de oferta em UBS com pelo menos uma equipe da ESF também é coincidente com estudos realizados nas últimas duas décadas, em diferentes regiões do país ^23^ ^,^ ^24^. O efeito da ESF também está expresso na associação da completude das ações com a presença de quatro ou mais ACS na UBS, na utilização de critério de vulnerabilidade e risco para cadastramento populacional, e na disponibilidade de conselho local de saúde ativo. A relevância do modelo da ESF na qualificação do cuidado tem sido associada à adscrição territorial, ao vínculo com a comunidade e à participação social, ao acompanhamento longitudinal dos usuários e à implementação de ações de promoção, prevenção e controle de condições crônicas de saúde ^44^. Logo, os resultados indicam a capacidade da ESF em articular e manejar intervenções individuais e comunitárias de forma integral e equitativa, mediante busca ativa, acompanhamento sistemático e contínuo de pessoas com condições crônicas, uso de protocolos clínicos e coordenação com outros níveis de atenção ^45^ ^,^ ^46^. Diversos estudos apontam que maiores coberturas municipais da ESF estão associadas a melhores desfechos em saúde, como redução de hospitalizações por condições sensíveis à APS ^47^ ^,^ ^48^.

A completude da oferta de cuidado associou-se positivamente ao apoio de eMulti, resultado consistente com a literatura, que destaca a importância da abordagem interdisciplinar para o manejo efetivo das condições crônicas ^49^ ^,^ ^50^. O desempenho positivo das UBS com eMulti pode ser explicado pela ampliação do apoio matricial e pelo fortalecimento do trabalho colaborativo entre as equipes, aspectos fundamentais para a atenção integral e longitudinal, reforçando o papel da APS como coordenadora do cuidado e ordenadora da rede de atenção. O apoio matricial vai além das reuniões periódicas e envolve processos de educação permanente, supervisão técnica e assistência compartilhada, integrando saberes e práticas e assim possibilitando às equipes mais recursos para lidar com situações complexas ^51^ ^,^ ^52^. Entretanto, é importante reconhecer que a presença de eMulti na APS ainda é insuficiente para atender à crescente demanda das condições crônicas.

As UBS com profissionais inseridos em educação permanente e aquelas com profissionais especializados em MFC e em enfermagem em saúde da família apresentaram maior completude das ações. A literatura aponta que a educação permanente, ao articular reflexão crítica sobre o processo de trabalho e a atualização técnica contínua, contribui para o fortalecimento de práticas clínicas, a ampliação da capacidade resolutiva e a incorporação de abordagens centradas no autocuidado apoiado e na gestão de risco ^53^. Experiências exitosas mostram que a consolidação da educação permanente na rotina das UBS resulta em maior adesão dos usuários, menor absenteísmo e melhor coordenação do cuidado ^54^ ^,^ ^55^.

As evidências internacionais indicam que uma APS integral e abrangente, organizada longitudinalmente por equipes multiprofissionais, que compartilham o trabalho e são regularmente capacitadas, está associada a maiores benefícios para o cuidado de condições crônicas de saúde e à satisfação dos usuários. As barreiras à integralidade do cuidado são similares àquelas observadas neste estudo, com destaque para a fragilidade da infraestrutura dos serviços, a sobrecarga de trabalho das equipes, a fragmentação dos sistemas de informação, a escassez de meios diagnósticos e terapêuticos e os “gargalos”e longos tempos de espera para acesso a especialistas ^56^ ^,^ ^57^ ^,^ ^58^.

Um ponto de destaque em nossa abordagem foi a seleção das variáveis utilizadas para compor a completude da oferta. A escolha foi guiada por critérios baseados nas diretrizes vigentes, buscando representar ações recomendadas. O construto da completude da oferta de ações foi revelador dos desafios persistentes para garantir cuidado integral a pessoas com hipertensão, diabetes e obesidade na APS. Entretanto, essa também indica fragilidade, pois parte de ações importantes para o cuidado integral das três condições estudadas foi contemplada apenas para uma das condições, a exemplo da aferição da pressão arterial somente para pessoas com hipertensão e não para pessoas com diabetes ou obesidade.

O artigo analisa exclusivamente dados do Censo das UBS compartilhando suas fortalezas e debilidades. Nesta perspectiva, as fragilidades de um levantamento censitário online afetam o manuscrito. Ressaltamos a possibilidade de viés de informação, uma vez que houve a indicação dos respondentes pelos gestores, o que pode ter levado a uma imprecisão das respostas. Porém, não foram observadas limitações relativas ao delineamento, à inclusão das UBS e às análises. Além disso, é preciso sublinhar a modelagem multinível adotada, que considera a estrutura hierárquica dos dados, produzindo estimativas mais concretas e, após o ajuste, destacar a contribuição de cada característica do contexto e das UBS para a completude da oferta. O ICC ≥ 0,80 indica uma forte dependência das UBS do contexto municipal, cujas características modelam as desigualdades observadas na completude da oferta de atenção.

O presente estudo evidencia a importância de aprofundar a investigação da tríade de agravos, considerando o crescimento das DCNT e de sua ocorrência simultânea. Entre esses agravos, a completude da oferta de cuidados à obesidade destaca-se como um desafio sistêmico. Futuros estudos orientados para a triangulação de informações, integração de novas bases de dados e aprofundamento da análise da qualidade e efetividade das ações na APS, poderão aperfeiçoar o monitoramento e subsidiar políticas públicas mais responsivas. A continuidade dos processos avaliativos é essencial, especialmente quando associada a investigações presenciais que incorporem as perspectivas de usuários e profissionais de saúde, fortalecendo a compreensão da relação entre oferta, acesso e efetividade das ações de cuidado. Os resultados convergem para a necessidade de fortalecimento estrutural e organizacional da APS em todo o território nacional, com aumento e reorientação do financiamento público, incluindo as boas práticas na ampliação dos indicadores de desempenho das equipes de APS, em sintonia com as ações de saúde contempladas no presente estudo, a qualificação permanente das equipes e o aperfeiçoamento dos processos de trabalho ^18^.

A região geopolítica, o porte populacional, o grau de vulnerabilidade social e a cobertura de ESF dos municípios interagiram com as características estruturais e organizacionais das UBS, influenciando significativamente a completude das ações. Os municípios com maior cobertura da ESF e maior vulnerabilidade social apresentaram melhores padrões de oferta, evidenciando o papel protetor e equitativo do modelo da ESF. O fortalecimento da ESF e a consolidação do trabalho multiprofissional têm potencial para reduzir desigualdades históricas, ampliar o acesso, garantir a integralidade do cuidado e elevar a resolutividade da APS, consolidando-a como pilar fundamental da efetividade e da equidade do SUS.

## Data Availability

As fontes de informação utilizadas no estudo estão indicadas no corpo do artigo.
